# Initial Clinical Experience with Percutaneous Endocardial Septal Pulsed Field Ablation in Hypertrophic Obstructive Cardiomyopathy: A First-in-Human Case Series

**DOI:** 10.3390/jcm15176620

**Published:** 2026-08-27

**Authors:** Haoruo Zhang, Yanfeng Tian, Songqing Yang, Rui Cao, Haoran Jing, Chunlin Gong, Jiaoyue Zhong, Yuran Chen, Siting Hong, Zhaojun Wang

**Affiliations:** 1 Department of Cardiology, First Affiliated Hospital, Harbin Medical University, Harbin 150001, China; 2 Department of Cardiology, Second Affiliated Hospital, Nanchang University, Nanchang 330006, China

**Keywords:** hypertrophic obstructive cardiomyopathy, pulsed field ablation, septal reduction therapy, left ventricular outflow tract obstruction, catheter ablation

## Abstract

**Background:** Pulsed field ablation is a nonthermal ablation modality with a potentially favorable tissue selectivity profile. However, its application in septal reduction therapy for hypertrophic obstructive cardiomyopathy remains unexplored clinically. We investigated the feasibility and short-term outcomes of percutaneous endocardial septal pulsed field ablation in patients with symptomatic hypertrophic obstructive cardiomyopathy. **Methods:** Five patients with symptomatic hypertrophic obstructive cardiomyopathy and persistent left ventricular outflow tract obstruction despite optimized conventional medical therapy underwent percutaneous endocardial septal pulsed field ablation guided by intracardiac echocardiography and 3-dimensional electroanatomical mapping. **Results:** Two-month follow-up data were available for all patients. Mean resting and provoked left ventricular outflow tract gradients decreased from 60.20 ± 14.10 and 93.60 ± 17.73 mmHg at baseline to 20.60 ± 5.18 and 27.80 ± 9.37 mmHg at 2 months, respectively. Improvements were also observed in systolic anterior motion, symptoms, 6 min walk distance, and Kansas City Cardiomyopathy Questionnaire score. One patient developed transient left bundle branch block and another transient complete atrioventricular block during the procedure; neither persisted. No persistent major procedure-related complications were observed during follow-up. **Conclusions:** In this small first-in-human case series, percutaneous endocardial septal pulsed field ablation was feasible and was associated with favorable short-term hemodynamic and clinical changes in selected patients with symptomatic hypertrophic obstructive cardiomyopathy. These findings are hypothesis-generating and require confirmation in larger studies with longer follow-up.

## 1. Introduction

Hypertrophic cardiomyopathy (HCM) is a common inherited cardiac disease, and left ventricular outflow tract obstruction (LVOTO) is a major determinant of symptoms and prognosis in a substantial subset of patients [1,2]. In patients with persistent symptoms despite guideline-directed medical therapy, septal reduction therapy (SRT) is an established treatment option [3]. Surgical septal myectomy and alcohol septal ablation (ASA) are established SRT options; however, their applicability may be constrained in selected patients by procedural invasiveness, septal perforator anatomy, institutional expertise, or the risk of conduction disturbance [4,5].

Percutaneous endocardial septal ablation (PESA) using radiofrequency energy has emerged as an alternative catheter-based approach, particularly in patients who are not ideal candidates for surgery or ASA. This strategy avoids reliance on septal coronary anatomy and allows endocardial targeting of the hypertrophied septum. However, concerns remain regarding lesion predictability, periprocedural myocardial edema with possible transient aggravation of obstruction, and conduction injury [6,7]. Accordingly, there remains an unmet need for a catheter-based septal reduction technique with improved controllability and a potentially more favorable safety profile.

Pulsed field ablation (PFA) is a predominantly nonthermal ablation modality based on irreversible electroporation and has shown a potentially favorable tissue selectivity profile in preclinical and early clinical settings [8,9]. Clinical experience in atrial fibrillation and selected ventricular arrhythmias provides a rationale for exploring PFA in septal reduction near adjacent critical structures [10,11]. However, its safety, lesion characteristics, and clinical utility for septal reduction in hypertrophic obstructive cardiomyopathy have not been established. We therefore performed an initial clinical evaluation of percutaneous endocardial septal pulsed field ablation (PES-PFA) in patients with symptomatic hypertrophic obstructive cardiomyopathy (HOCM) and persistent LVOTO despite optimized conventional medical therapy, focusing primarily on procedural feasibility and short-term safety, with exploratory assessment of early hemodynamic and clinical outcomes.

## 2. Materials and Methods

### 2.1. Participants

Between July and December 2025, 68 patients with symptomatic HOCM and an inadequate response to conventional medical therapy were evaluated. Of these, 52 pursued alternative management, whereas 16 were formally assessed for PES-PFA eligibility; 11 were excluded according to the prespecified criteria, and 5 underwent PES-PFA (Supplemental Figure S1). An inadequate response to conventional medical therapy was defined as persistent symptoms with an LVOT gradient ≥30 mmHg despite optimized high-dose or individually maximally tolerated conventional negative inotropic therapy with a beta-blocker and/or a nondihydropyridine calcium channel blocker. Key inclusion criteria were age 18–80 years, septal thickness ≥13 mm, and either an LVOT gradient ≥50 mmHg at rest or with provocation or a resting gradient ≥30 mmHg accompanied by significant exertional symptoms. Patients with non-obstructive HCM or with clinical or imaging findings that precluded safe or appropriate PES-PFA were excluded; detailed eligibility criteria are provided in the Supplementary Materials.

All 5 patients underwent a multidisciplinary assessment for established SRT options. Patients 1 and 2 declined surgical myectomy because of concerns about surgical invasiveness and preferred a less invasive approach, whereas Patient 3 declined because of concerns about operative trauma and conduction system injury. Patient 4, who had concomitant cerebrovascular disease and increased perioperative neurologic and anesthetic risk, also declined surgery. Patient 5 was considered unsuitable for either surgical myectomy or ASA because pre-existing left bundle branch block (LBBB) conferred a high risk of clinically significant conduction block and concomitant heart failure with reduced ejection fraction increased the overall procedural risk. ASA was not performed in Patients 1, 2, and 4 because no suitable target septal perforator was identified, and in Patient 3 because severe tortuosity of the target septal perforator precluded stable balloon positioning and occlusion. Patient-level medication regimens, treatment response or dose limitations, and reasons for not undergoing surgical myectomy or ASA are summarized in Supplemental Table S5.

The study was conducted in accordance with the Declaration of Helsinki and was approved by the institutional ethics committee of the First Affiliated Hospital of Harbin Medical University (approval No. 2024XJSS11). At the time of the procedures, the AForcePlus catheter had been approved by the National Medical Products Administration of China for adjunctive ablation as part of a PFA system for drug refractory, recurrent, symptomatic paroxysmal atrial fibrillation. Its use for ventricular septal ablation was off-label; this use was reviewed and approved by the institutional ethics committee as part of the study protocol. The study was registered at the Chinese Clinical Trial Registry (ChiCTR2500104570), and all patients provided written informed consent.

The primary objectives were procedural feasibility and periprocedural safety, whereas key exploratory outcomes included changes in resting and provoked LVOT gradients, New York Heart Association (NYHA) functional class, 6 min walk distance, Kansas City Cardiomyopathy Questionnaire (KCCQ) score, and imaging findings at 2-month follow-up.

### 2.2. Imaging Assessment

Transthoracic echocardiography (TTE) was performed before the procedure, immediately after the procedure, and at 2-month follow-up. Provoked LVOT gradients were assessed in all patients using a standardized, target-directed Valsalva maneuver at baseline, immediately after the procedure, and at 2-month follow-up. With the patient in the left lateral decubitus position, a modified sphygmomanometer and tubing system was used to guide the patient to maintain an expiratory pressure of >40 mmHg for >10 s [12]. The peak LVOT gradient was measured by continuous-wave Doppler during the sustained strain phase (phase II) of the Valsalva maneuver. The same provocation method was used for each patient at all 3 assessment time points. Measurements were averaged over 3 consecutive cardiac cycles, or at least 5 cycles in patients with atrial fibrillation [13]. Abnormal chordal-related systolic anterior motion (SAM) was recorded when present. Primary echocardiographic measures were resting and provoked LVOT gradients; other measures included septal thickness, mitral valve SAM, left ventricular ejection fraction (LVEF), left ventricular posterior wall thickness at end-diastole (LVPWd), and left ventricular end-diastolic diameter (LVEDD). Mitral regurgitation severity was graded using an integrated echocardiographic assessment. Cardiac magnetic resonance (CMR) with late gadolinium enhancement was performed before PES-PFA and at 2 months to assess post-ablation septal enhancement.

### 2.3. Electrocardiographic and Laboratory Assessments

All patients underwent 24 h ambulatory electrocardiogram (ECG) monitoring before the procedure to characterize baseline rhythm and identify additional arrhythmias or conduction abnormalities. Standard 12-lead ECGs were obtained at baseline, immediately after completion of the index procedure, on postprocedural day 1, and at 2-month follow-up to assess changes in cardiac rhythm and conduction. PR intervals were assessed on ECG tracings recorded during sinus rhythm. All patients underwent continuous telemetry monitoring for 48 h after the procedure. At 2-month follow-up, all patients underwent standard 12-lead ECG; Patients 1, 4, and 5 also underwent additional 24 h Holter monitoring. Holter monitoring was performed in Patients 1 and 5 because of intraprocedural conduction disturbances and in Patients 4 and 5 because they had undergone pulmonary vein isolation (PVI). Peripheral venous blood samples were obtained at baseline, on postprocedural day 1, and at 2-month follow-up for complete blood count, biochemical testing, and measurement of cardiac injury biomarkers to assess periprocedural hematologic changes, myocardial injury, and hepatic or renal dysfunction. Detailed electrocardiographic acquisition and laboratory assay methods are provided in the Supplementary Materials.

### 2.4. PFA System and Energy Delivery

PES-PFA was performed using a 7.5-F, non-irrigated, magnetically tracked, contact force-sensing quadripolar focal PFA catheter (AForcePlus^TM^, model 1552003SD08), a dedicated PFA generator (PFG-micro1), and a 3-dimensional electroanatomical mapping system (HT Viewer; all devices from APT Medical, Shenzhen, China). The catheter incorporated one 2 mm tip electrode and three 2 mm ring electrodes, with interelectrode spacing of 3–3–3 mm. All four electrodes could be used for intracardiac electrogram recording, electrophysiological mapping, and pacing. Symmetric biphasic, microsecond-scale pulses were delivered in a bipolar configuration between the tip electrode and the most distal ring electrode. The system provided three preset output voltage levels: 800, 1600, and 1800 V. All septal PFA applications were delivered with R-wave synchronization enabled to avoid pulse delivery during the ventricular relative refractory period. The exact pulse width, interphase delay, number of pulses per train, and pulse repetition frequency within each train were proprietary parameters that were not made available to the investigators by the manufacturer.

### 2.5. Procedural Technique

**Figure 1:**
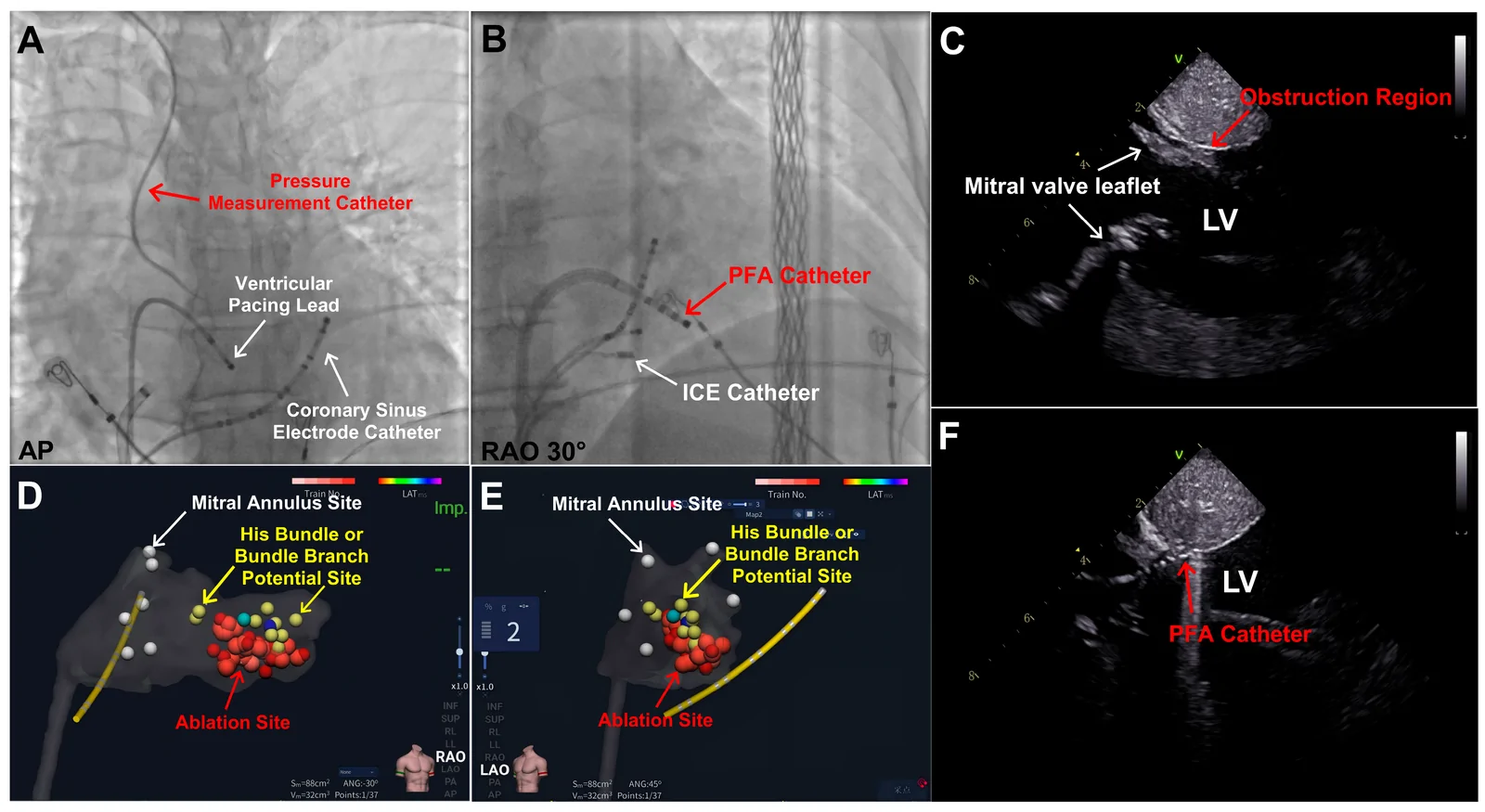
Procedural illustration of pulsed field ablation (PFA) for hypertrophic obstructive cardiomyopathy guided by fluoroscopy, 3-dimensional electroanatomic mapping, and intracardiac echocardiography (ICE). (**A**) Anteroposterior fluoroscopic view during pressure measurement, showing the pressure measurement catheter (red arrow), ventricular pacing lead, and coronary sinus electrode catheter (white arrow). (**B**) Fluoroscopic image obtained immediately before ablation, showing the PFA catheter (red arrow) and ICE catheter (white arrow). (**C**) ICE image obtained before ablation, showing the obstructive region (red arrow), mitral valve leaflet (white arrow), and systolic anterior motion. (**D**,**E**) Three-dimensional electroanatomic maps obtained during ablation. White points indicate the mitral annulus, yellow points indicate the His bundle or bundle branch region, and red points indicate the ablation sites. (**F**) ICE image obtained during ablation, showing good apposition of the PFA catheter to the interventricular septum.

Written informed consent for the procedure was obtained from all patients before intervention. A temporary pacing lead was positioned at the right ventricular apex for backup pacing, and a decapolar catheter was positioned in the coronary sinus to monitor intraprocedural conduction changes. A 7-F angiographic catheter was advanced retrogradely into the left ventricle through the radial artery. At each assessment, left ventricular and supravalvular aortic pressures were recorded using a sequential pullback technique, and the transaortic pressure gradient was calculated. Measurements were obtained before PES-PFA, repeated after each energy delivery, and repeated at the completion of PES-PFA (Figure 1A).

The same pressure-recording system and measurement protocol were used in all patients, and the system was calibrated before each measurement. All patients remained in sinus rhythm and breathed spontaneously during PES-PFA. Local infiltration anesthesia was used only for vascular access; the procedure was otherwise performed without sedation or general anesthesia. No vasoactive medication was administered, and no fluid bolus or other intervention intended to alter loading conditions was given between measurements. Pressure values were obtained from multiple consecutive stable beats; recordings during premature ventricular contractions, ventricular tachycardia, or postextrasystolic beats were excluded.

Before PES-PFA, Patients 1–4 underwent right ventricular septal endomyocardial biopsy (EMB) as an exploratory adjunct to the noninvasive evaluation, including CMR, to assess for infiltrative or storage cardiomyopathy phenocopies and to characterize the baseline histopathological features of the interventricular septal myocardium (Supplemental Figure S2). The biopsy procedure and its potential risks were discussed with the patients during the preprocedural informed consent process, and written informed consent was obtained. Patient 5 had pre-existing LBBB, with a PR interval of 192 ms and a QRS duration of 192 ms, but no atrioventricular block, as well as paroxysmal atrial fibrillation requiring PVI. After multidisciplinary assessment, isolated LBBB with preserved atrioventricular conduction was not considered an absolute contraindication to enrollment. The planned PES-PFA and PVI were performed from left-sided endocardial structures and did not involve direct instrumentation of the right ventricular septum or intentional targeting of the right bundle branch. Nevertheless, right ventricular septal EMB was not performed in Patient 5 because injury to the functionally intact right bundle branch during biopsy could precipitate complete atrioventricular block.

After transseptal access to the left atrium was established, the PFA catheter was advanced through the transseptal sheath into the left atrium and subsequently across the mitral valve into the left ventricle under fluoroscopic guidance (Figure 1B). The intracardiac echocardiography (ICE) catheter (SOUNDSTAR™, Biosense Webster, Irvine, CA, USA) was advanced across the tricuspid valve into the mid right ventricle and oriented toward the right ventricular apex (Figure 1B). After release of the initial anterior deflection, the catheter was gradually rotated clockwise, and leftward deflection was applied to bring the left ventricle into the imaging sector and to obtain a left ventricular long-axis view. Small adjustments in left/right deflection were then used to sweep the imaging plane across the basal and mid interventricular septum, allowing visualization of the hypertrophied obstructive septal segment and its relationship to the LVOT for ablation planning (Figure 1C). On the 3-dimensional map, the mitral annulus was marked with white tags, and the His-bundle and bundle branch regions were identified by electrophysiological mapping and marked with yellow tags (Figure 1D,E). Under ICE guidance, the ablation catheter was positioned in stable apposition to the basal and/or mid hypertrophied interventricular septum (Figure 1F) and completed ablation sites were marked with red tags on the 3-dimensional map (Figure 1D,E).

To quantify ablation coverage, the obstructive septal endocardial region was delineated on the reconstructed left ventricular geometry under ICE guidance. The transition between the hypertrophied obstructive septum and adjacent non-obstructive septal myocardium was identified in multiple ICE imaging planes. Corresponding boundary points were manually annotated with the PFA catheter on the 3-dimensional map and connected to form a closed contour, and the mapping system automatically calculated the enclosed endocardial surface area. After ablation, the outer boundary of the completed ablation tags was similarly contoured, and the corresponding ablated endocardial surface area was automatically calculated. Ablation coverage was defined as the ablated surface area divided by the predefined obstructive septal surface area and multiplied by 100%.

Before definitive ablation, test applications were delivered sequentially at each target site using low-dose pulsed field energy (800 V; 1 pulse train) followed by intermediate-dose energy (1600 V; 1 pulse train). Test ablation proceeded only when the catheter tip was at least 8 mm from the nearest His–bundle branch fibers and no P potential was recorded at the catheter tip. During test ablation, heart rate, rhythm, QRS morphology, and atrioventricular conduction parameters, including the A–V and H–V intervals, were continuously monitored. If no significant changes in QRS morphology or atrioventricular conduction were observed, definitive ablation was delivered using high-dose pulsed field energy (1800 V; 5 pulse trains), with a contact force of 10–15 g and a lesion duration of 5 s.

If ventricular tachycardia lasting >2 s or frequent premature ventricular contractions occurred during a test application, energy delivery at that site was immediately terminated and ablation was redirected to an adjacent site. If paradoxical elevation of the transaortic pressure gradient, new atrioventricular block, or new bundle branch block occurred after a test application, further ablation in that region was discontinued and the procedure was temporarily suspended for observation. If complete or high-grade atrioventricular block occurred, intravenous dexamethasone was administered to facilitate recovery of atrioventricular conduction, with temporary right ventricular apical pacing provided as needed. Ablation was avoided when the intended hypertrophied septal target overlapped with a mapped conduction system region.

Procedural endpoints were defined as either >50% ablation coverage of the predefined obstructive septal endocardial surface or a >50% reduction in the invasive transaortic pressure gradient from baseline; if neither criterion was met, additional ablation was performed until at least 1 endpoint was achieved. After the PES-PFA portion of the index procedure had concluded and the final post-PES-PFA transaortic pressure gradient had been reassessed, PVI was performed in patients with concomitant atrial fibrillation requiring rhythm-control intervention according to standard clinical practice. Additional procedural details, including biopsy procedures, and intraprocedural supportive measures, are provided in the Supplementary Materials.

### 2.6. Statistical Analysis

Given the exploratory nature and small sample size of this 5-patient case series, no formal hypothesis testing was performed, and analyses were descriptive. Continuous variables are presented as mean ± SD and categorical variables as counts or percentages. All enrolled patients completed the 2-month follow-up, and no outcome data were missing.

## 3. Results

### 3.1. Baseline Characteristics

Baseline characteristics are summarized in Table 1, with individual data provided in Supplemental Tables S1–S5. The mean age was 54.20 ± 10.62 years, and all five patients were male; four were in NYHA functional class III. Mean resting and provoked LVOT gradients were 60.20 ± 14.10 and 93.60 ± 17.73 mmHg, respectively, and mean end-diastolic septal thickness was 21.20 ± 4.83 mm. LVOT obstruction was confined to the basal septum in two patients and extended from the basal-to-mid septum in three, with SAM present in all patients. Pathogenic variants were identified in two patients (MYBPC3 and MYH7, 1 each).

### 3.2. Procedural Characteristics and Periprocedural Complications

Procedural characteristics and 60-day complications are summarized in Table 2, with individual data provided in Supplemental Table S6. PES-PFA was performed in all five patients via a transseptal approach. EMB was successfully performed before PES-PFA in Patients 1–4. Histopathological findings were consistent with HCM and revealed no clinically actionable alternative diagnosis. Congo red staining with polarized-light examination showed no amyloid deposition in the sampled myocardium, and periodic acid–Schiff staining showed no overt morphological evidence of a storage cardiomyopathy. The biopsy findings did not alter clinical diagnosis, pharmacological treatment, ablation strategy, or follow-up plan, and no biopsy-related complications occurred.

PES-PFA was delivered using preset output voltages of 800, 1600, and 1800 V. The mean number of completed septal ablation sites was 29.40 ± 6.62, and the mean PES-PFA energy delivery time was 147.00 ± 33.09 s. By left heart catheterization, the mean transaortic pressure gradient decreased from 90.80 ± 20.90 mmHg before PES-PFA to 40.20 ± 16.72 mmHg after PES-PFA.

Transient conduction disturbances occurred in two patients during PES-PFA. In Patient 1, during definitive ablation at the 29th target site using a preset output voltage of 1800 V with five planned pulse trains, acute QRS widening consistent with LBBB occurred after delivery of the first pulse train. Energy delivery was immediately discontinued. QRS narrowing began approximately 2 min after cessation of ablation, and the baseline QRS morphology was fully restored by 3 min without temporary pacing; further septal ablation was then terminated (Supplemental Figure S3). Patient 5 had pre-existing LBBB, with a PR interval of 192 ms and a QRS duration of 192 ms, but no baseline atrioventricular block. At the 23rd target site, the low-dose test application at 800 V with one pulse train was followed by two ventricular pacing signals, after which intrinsic atrioventricular conduction resumed without persistent disturbance. The subsequent intermediate-dose test application at the same site at 1600 V with one pulse train immediately induced complete atrioventricular block, requiring ventricular pacing for approximately 4 min. Atrioventricular conduction recovered approximately 4 min after onset following withdrawal of the ablation catheter and intravenous dexamethasone administration, and further septal ablation was terminated (Supplemental Figure S4).

After the PES-PFA portion of the index procedure had concluded and the final post–PES-PFA transaortic pressure gradient had been reassessed, Patients 4 and 5, both of whom had paroxysmal atrial fibrillation, subsequently underwent PVI. In both patients, the temporary pacing lead was retained during PVI. In all patients, the temporary pacing lead was removed after completion of the entire index procedure. The reported procedural time represented the overall duration of the index procedure and included the concomitant procedures. Although transient conduction disturbances occurred in two patients, no persistent conduction block or other major procedure-related complications occurred through 60 days, and no patient required permanent pacemaker implantation (Table 2).

### 3.3. Clinical and Functional Findings

Detailed data are provided in Supplemental Tables S7 and S9. By postprocedural day 1, three patients reported less chest pain. At 2 months, four patients reported less chest pain, whereas one reported little change. At 2-month follow-up, NYHA functional class was II in four patients and III in one, compared with class II in one and class III in four at baseline.

### 3.4. Changes in CMR Imaging from Baseline to 2 Months

**Figure 2:**
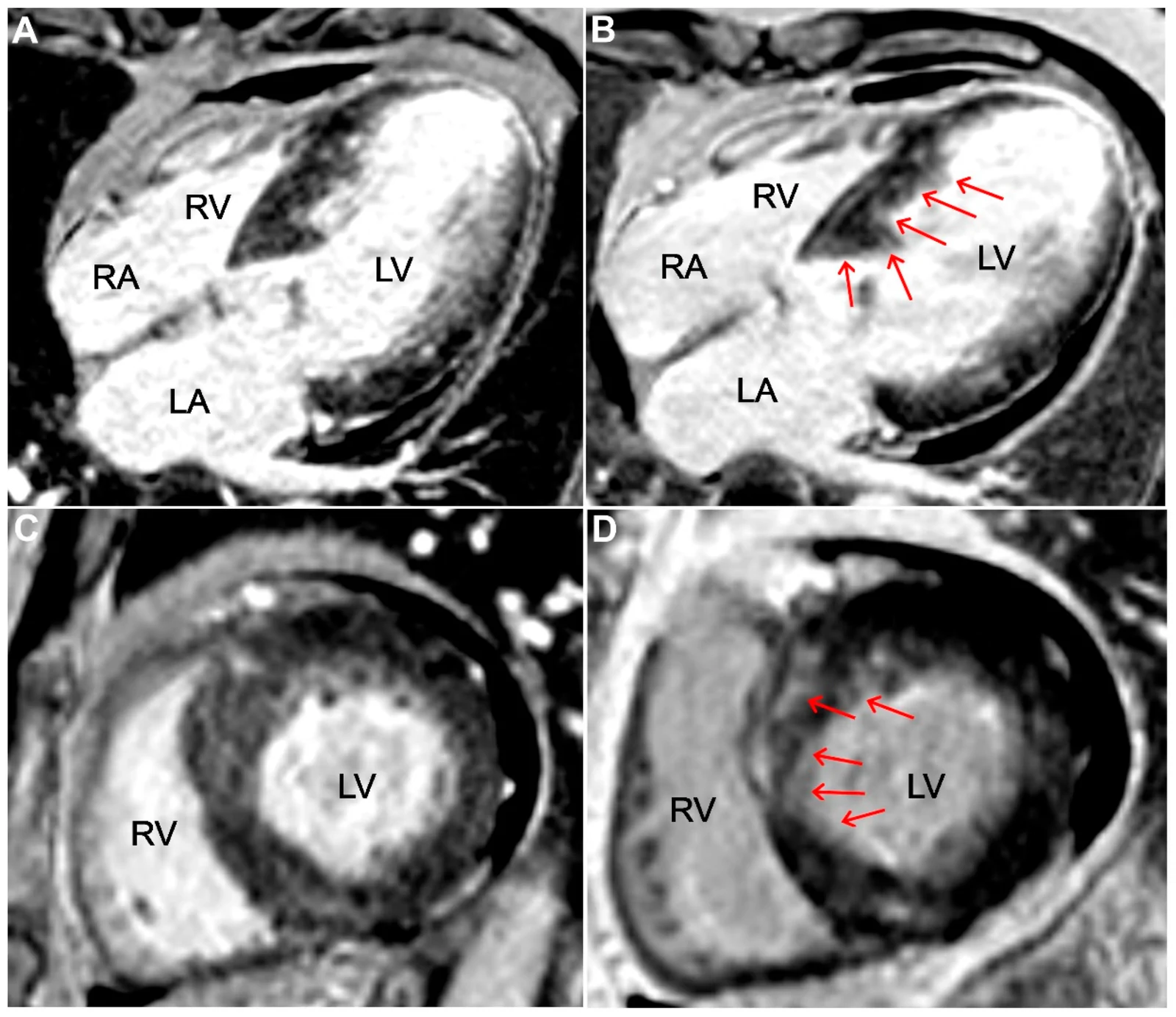
Cardiac magnetic resonance late gadolinium enhancement images before pulsed field ablation and at 2-month follow-up. Preprocedural 4-chamber and short-axis images are shown in (**A**) and (**C**), respectively. At 2 months, the corresponding 4-chamber (**B**) and short-axis (**D**) images show a new focal area of late gadolinium enhancement on the left ventricular aspect of the basal-to-mid interventricular septum near the left ventricular outflow tract (red arrows). This lesion was not present before ablation and was spatially concordant with the ablation target region, consistent with localized postprocedural myocardial injury within the targeted septum.

At 2 months after the procedure, late gadolinium enhancement imaging demonstrated new focal enhancement along the LV side of the basal-to-mid interventricular septum, adjacent to the LVOT. The location of this lesion was concordant on both the four-chamber and left ventricle short-axis views (Figure 2B,D). Compared with baseline images (Figure 2A,C), this represented a new postprocedural finding. Its distribution largely corresponded to the PES-PFA target region, consistent with localized postprocedural myocardial injury within the targeted septum.

### 3.5. Periprocedural and 2-Month Echocardiographic, 6-Minute Walk Test, and KCCQ Findings

**Figure 3:**
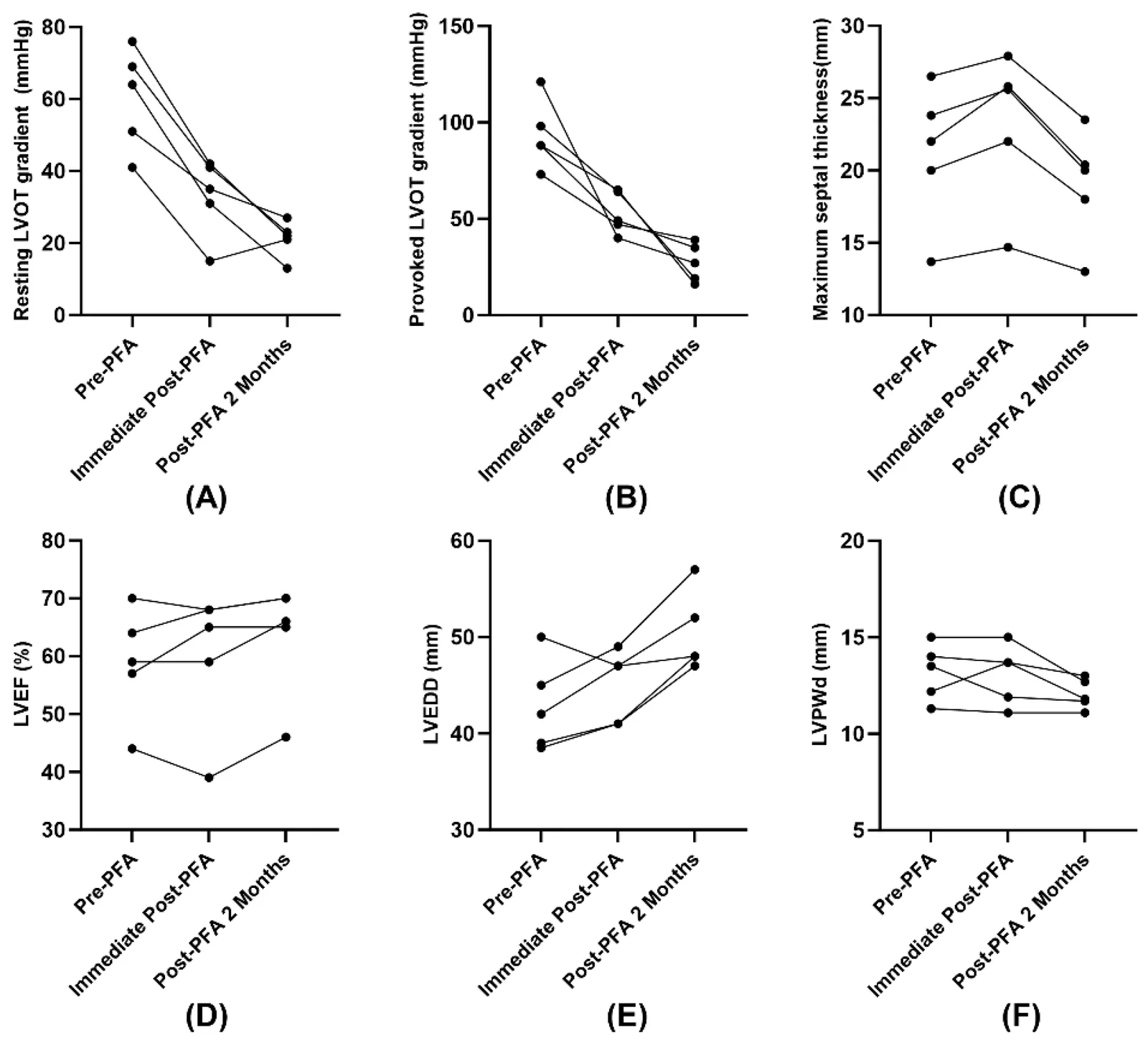
Periprocedural and 2-month changes in echocardiographic parameters in patients with hypertrophic obstructive cardiomyopathy. (**A**) Resting left ventricular outflow tract (LVOT) gradient. (**B**) Provoked LVOT gradient. (**C**) Maximum septal thickness. (**D**) Left ventricular ejection fraction (LVEF). (**E**) Left ventricular end-diastolic diameter (LVEDD). (**F**) Left ventricular posterior wall thickness at end-diastole (LVPWd). Each line represents an individual patient. Measurements were obtained at baseline, immediately after completion of the entire index procedure, including pulmonary vein isolation when performed, and at 2-month follow-up.

Echocardiographic findings are summarized in Table 3 and Figure 3, with additional individual-level data provided in Supplemental Table S8. Resting LVOT gradient decreased from 60.20 ± 14.10 mmHg at baseline to 32.80 ± 10.92 mmHg immediately after the procedure and to 20.60 ± 5.18 mmHg at 2 months (Figure 3A). Provoked LVOT gradient decreased from 93.60 ± 17.73 mmHg to 53.00 ± 11.02 mmHg and then to 27.80 ± 9.37 mmHg, respectively (Figure 3B). Mild septal edema was observed immediately after the procedure, followed by a reduction in maximal septal thickness at 2 months. LVEF and LVPWd remained largely unchanged, whereas LVEDD increased over follow-up (Figure 3C–F).

SAM was present in all five patients at baseline, four immediately after the procedure, and two at 2 months. At baseline, mitral regurgitation was moderate in four patients and severe in one. Immediately after the procedure, mitral regurgitation improved in four patients and remained unchanged in one, with severity graded as mild in two patients, mild-to-moderate in one, moderate in one, and moderate-to-severe in one. At 2 months, mitral regurgitation was mild in three patients, mild-to-moderate in one, and moderate in one, representing improvement from baseline in all patients. Compared with the immediate postprocedural assessment, mitral regurgitation further improved in two patient and remained stable in the other three, with no worsening observed (Supplemental Table S8).

**Figure 4:**
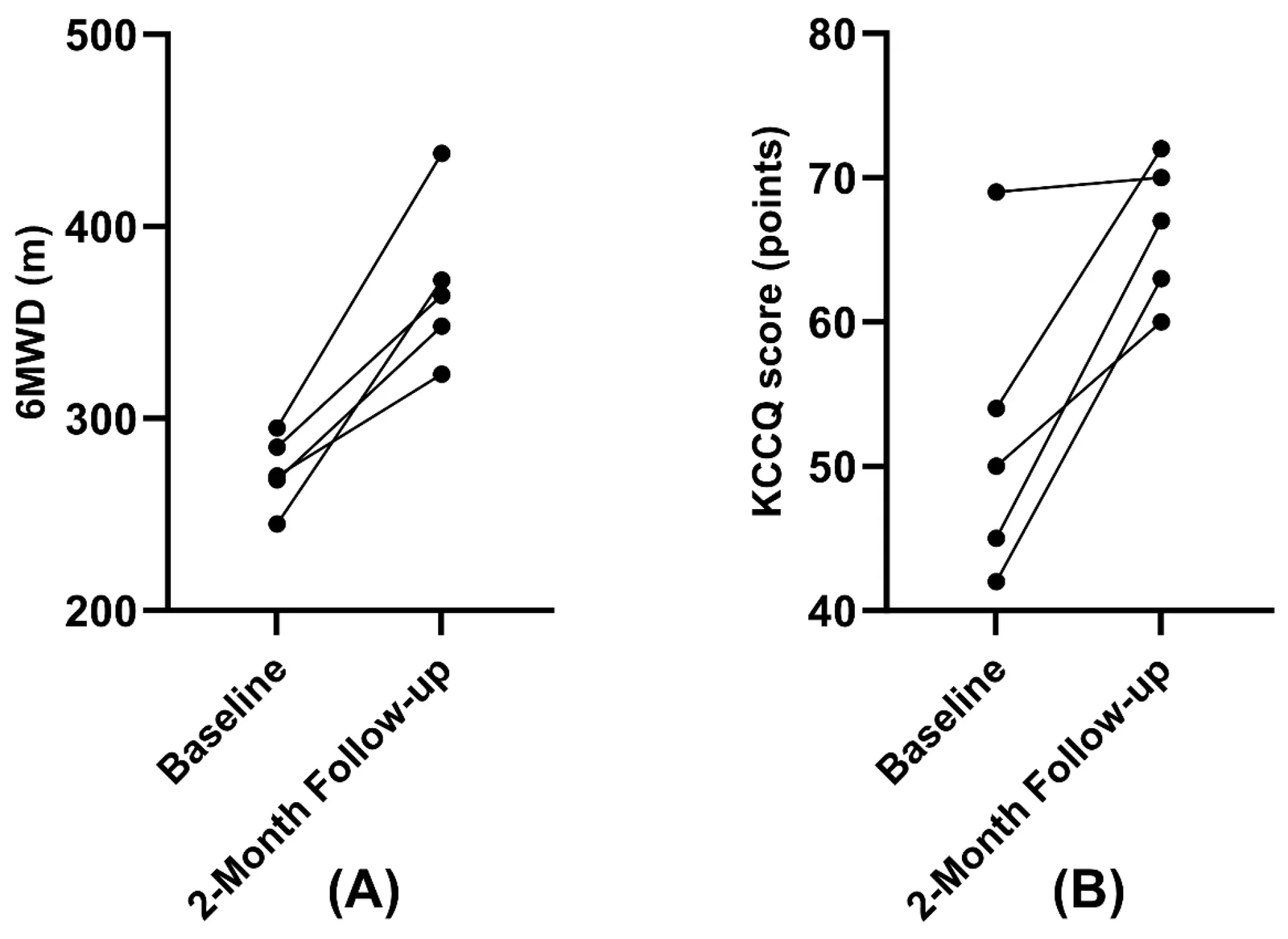
Changes in 6 min walk distance and Kansas City Cardiomyopathy Questionnaire score from baseline to 2-month follow-up. (**A**) Six-minute walk distance (6MWD). (**B**) Kansas City Cardiomyopathy Questionnaire (KCCQ) overall score. Each line represents an individual patient. Assessments were performed at baseline and at 2-month follow-up after the index procedure.

Six-minute walk test and KCCQ findings are shown in Table 3 and Figure 4, with detailed individual data provided in Supplemental Table S9. At 2 months, 6 min walk distance increased from 272.60 ± 19.01 m to 369.00 ± 42.87 m (Figure 4A), and KCCQ score increased from 52.00 ± 10.56 to 66.40 ± 4.93 (Figure 4B).

### 3.6. Periprocedural and 2-Month Electrocardiographic Findings

**Figure 5:**
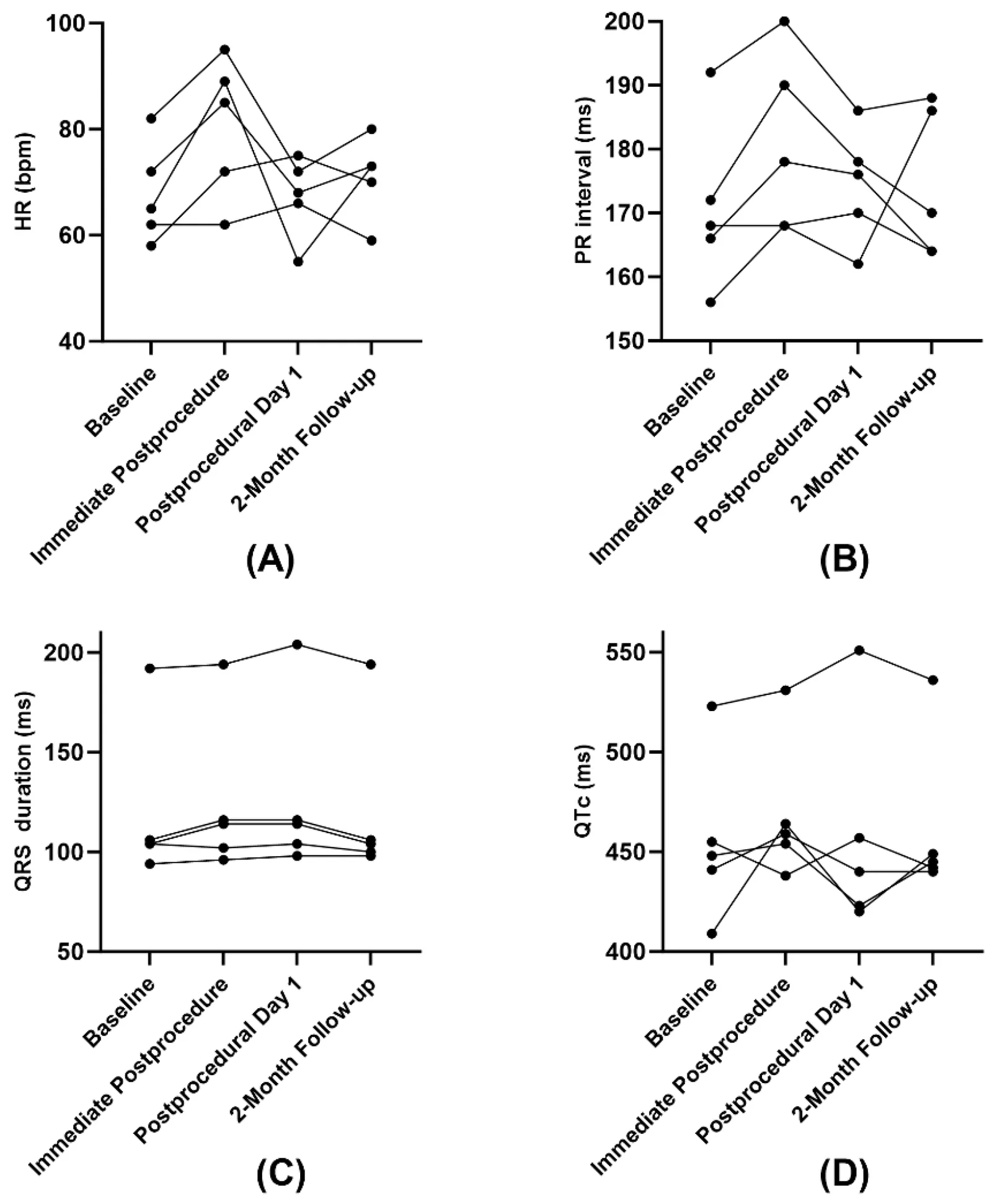
Periprocedural and 2-month changes in electrocardiographic parameters in patients with hypertrophic obstructive cardiomyopathy. (**A**) Heart rate (HR). (**B**) PR interval. (**C**) QRS duration. (**D**) Corrected QT interval (QTc). Each line represents an individual patient. Standard 12-lead electrocardiographic measurements were obtained at baseline, immediately after completion of the index procedure, on postprocedural day 1, and at 2-month follow-up.

ECG parameters are shown in Figure 5 and Supplemental Table S13, with detailed individual data provided in Supplemental Table S10. Heart rate and corrected QT interval (QTc) remained largely unchanged throughout the periprocedural period and 2-month follow-up (Figure 5A,D). PR interval showed mild immediate postprocedural prolongation but returned to near-baseline values by postprocedural day 1 and remained stable at 2 months (Figure 5B). QRS duration increased modestly immediately after the procedure and on postprocedural day 1, with recovery toward baseline levels at 2 months (Figure 5C). During 48 h postprocedural telemetry, no recurrent high-grade atrioventricular block, new bundle branch block, or symptomatic bradycardia was detected. At 2-month follow-up, no delayed high-grade atrioventricular block or other new conduction abnormalities were detected on standard 12-lead ECGs obtained in all patients or on additional 24 h Holter monitoring performed in Patients 1, 4, and 5. No atrial fibrillation was detected on the 2-month Holter recordings in Patients 4 and 5, and no patient required permanent pacemaker implantation.

### 3.7. Periprocedural and 2-Month Hematologic and Biochemical Findings

Complete blood count results are summarized in Supplemental Table S14 and Figure S5, with detailed individual data provided in Supplemental Table S11. Mean red blood cell count and hemoglobin decreased slightly on postprocedural day 1 and returned toward baseline levels by 2 months (Supplemental Figure S5A,B). White blood cell count, neutrophil count, and neutrophil percentage increased transiently on postprocedural day 1 and returned toward baseline levels by 2 months (Supplemental Figure S5C–E). Platelet count remained largely unchanged (Supplemental Figure S5F).

Biochemical findings are shown in Supplemental Table S14 and Figure S6, with detailed individual data provided in Supplemental Table S12. Cardiac troponin I (cTnI) and creatine kinase-MB (CK-MB) increased markedly on postprocedural day 1 and declined substantially toward baseline levels by 2 months (Supplemental Figure S6A,B). Total, indirect, and direct bilirubin also increased modestly on postprocedural day 1 and returned to baseline by 2 months (Supplemental Figure S6C–E). Mean serum creatinine remained largely stable, with no clinically significant renal dysfunction observed (Supplemental Figure S6F).

## 4. Discussion

In this first-in-human case series, PES-PFA was feasible in five patients with symptomatic HOCM with persistent LVOTO despite optimized conventional medical therapy, and was associated with favorable short-term reductions in LVOTO, accompanied by improvements in symptoms, exercise capacity, and health status during 2-month follow-up. Transient conduction disturbances occurred in two patients, but no persistent major procedure-related complications were observed in this small cohort. These findings suggest that PES-PFA may represent an investigational catheter-based option for highly selected patients who are not suitable for, or decline, surgical myectomy or ASA; however, it should not be considered a replacement for established SRT approaches.

Cardiac myosin inhibitors (CMIs) have expanded therapeutic options for HOCM [14]. Nevertheless, SRT remains an established option for selected patients with symptomatic LVOTO when medical therapy is contraindicated, not tolerated, or insufficient to relieve symptoms or reduce clinically significant obstruction [2]. Although CMIs offer an important noninvasive treatment, their use may be limited by reduced systolic function, limited evidence in special populations, and the need for sustained adherence and monitoring [2,15]. Accordingly, CMIs and SRT should be viewed as complementary components of individualized management rather than competing approaches [15].

Endocardial septal radiofrequency ablation was introduced as a catheter-based alternative septal reduction approach in 2004 [16]. The technique was subsequently refined with intracardiac echocardiography and 3-dimensional electroanatomical mapping guidance [6,17]. These developments established the feasibility of PESA, particularly in patients in whom conventional septal reduction strategies are limited by septal perforator anatomy or procedural preference. However, because lesion formation depends on thermal injury, radiofrequency-based septal ablation may be associated with less controllable lesion extent, transient edema-related worsening of outflow tract obstruction, and collateral injury to adjacent structures, including the conduction system [6,7,17]. Accordingly, although prior PESA series have demonstrated proof of concept, important procedural and safety considerations remain, providing the rationale for exploring alternative energy sources, including nonthermal approaches, for septal reduction.

PFA produces myocardial injury predominantly through irreversible electroporation rather than thermal conduction and is threshold-dependent and relatively tissue-selective, making it conceptually attractive for targeted septal reduction while minimizing collateral injury [18]. Preclinical ventricular studies have demonstrated well-demarcated lesions of sufficient depth and differential tissue sensitivity, including relative preservation of Purkinje fibers [19,20,21]; although PFA is not entirely nonthermal, its thermal component appears lower than that of radiofrequency ablation [22]. In this small series, mild septal edema and the absence of persistent conduction block were broadly consistent with these proposed properties, although these clinical observations do not establish mechanisms. Direct comparison with prior radiofrequency-based PESA is not possible because of major differences in study design and procedural methodology; the Cooper report was likewise an early five-patient experience, with 6-month follow-up available in four patients [6]. In that descriptive context, Cooper et al. reported delivery of radiofrequency energy for 28–42 min in a heat-based PESA setting; one patient developed edema-related paradoxical worsening of left ventricular outflow tract obstruction resulting in pulmonary edema, one patient died following significant retroperitoneal hemorrhage, and delayed complete heart block was also reported during follow-up [6]. By contrast, in the present PES-PFA series, the mean total energy delivery time was 147.00 ± 33.09 s across 29.40 ± 6.62 ablation sites, with mild septal edema and only transient conduction disturbances observed. These cross-study observations are descriptive and hypothesis-generating and do not establish comparative superiority in safety or efficacy; they support further evaluation of whether the lower thermal component of PFA may confer procedural advantages for septal reduction.

Beyond its mechanistic rationale, the clinical positioning of PES-PFA within the current landscape of SRT requires careful consideration. As with other emerging interventional technologies, PES-PFA has potential limitations that should be considered when selecting patients with HOCM for catheter-based septal reduction. Caution may be warranted in patients with advanced renal dysfunction because of potential hemolysis-related renal injury [23], in those with significant coronary artery disease or target septal regions adjacent to major coronary structures [24], and when ablation targets are located near critical conduction tissue where the safety margin remains incompletely defined. Accordingly, the relative roles of PESA and PES-PFA require further evaluation, and the choice of catheter-based septal reduction strategy should be individualized according to patient characteristics, septal anatomy, procedural objectives, and the evolving evidence base. In selected patients with concomitant arrhythmias requiring catheter-based electrophysiological interventions, combined procedures, including atrial fibrillation ablation or other indicated electrophysiological therapies, may be considered within the same procedural setting when clinically appropriate, although further evaluation of this approach is needed.

As with other SRT approaches, PES-PFA aims to selectively injure the septal myocardium to relieve LVOTO, although its energy source and mechanism of tissue injury differ from those of established approaches. Compared with chemical or thermal septal ablation, PFA is predominantly nonthermal, threshold-dependent, and relatively tissue-selective [18]. These characteristics suggest that the anti-obstructive effect of PES-PFA may evolve over time, with early functional suppression followed by later structural remodeling. In the short term, PFA may induce functional suppression of the targeted septal myocardium despite limited immediate anatomic change, resulting in early relief of LVOTO [25,26]. Over time, edema resolution and lesion remodeling may contribute to reduced septal thickness and further relief of LVOTO [26]. The observed reductions in LVOT gradient and improvements in functional status are consistent with this stage-dependent model but do not establish the underlying mechanisms. These mechanistic interpretations remain hypothetical and require validation in experimental studies and larger clinical cohorts with longer follow-up.

Although previous studies have suggested a relatively favorable and at least partly reversible conduction safety profile for PFA, our exploratory porcine observations and clinical findings indicate that its conduction effects may depend on the ablation site and extent of energy exposure. In the exploratory porcine experiments described in the Supplementary Note, repeated applications at an output voltage of 1800 V to the sinoatrial node and superior vena cava regions produced no obvious change in sinus rate or apparent sinoatrial node dysfunction (Supplemental Video S1), whereas a single application at the mapped His bundle region caused complete atrioventricular block that persisted throughout the 1 h observation period (Supplemental Video S2). Although these observations do not define specific safety thresholds, they indicate that high-voltage PFA delivered near the His bundle may cause serious and potentially persistent conduction disturbance. These observations informed a conservative clinical strategy incorporating focused para-Hisian mapping, separation from the mapped conduction system, stepwise energy escalation, continuous conduction monitoring, temporary pacing readiness, and immediate cessation of ablation when a new conduction abnormality occurred.

Despite these precautions, transient conduction disturbances occurred in two of five patients during PES-PFA and represent clinically important safety events in this small series. Patient 1 developed transient LBBB, whereas Patient 5, who had pre-existing LBBB but preserved atrioventricular conduction, developed transient complete atrioventricular block requiring temporary ventricular pacing; both abnormalities resolved within several minutes after energy delivery was stopped, and no further septal ablation was performed in either patient. No persistent or delayed high-grade atrioventricular block, new conduction abnormality, or symptomatic bradycardia was detected during 48 h telemetry or subsequent follow-up, and no patient required permanent pacemaker implantation. Mild transient prolongation of the PR interval and QRS duration generally returned toward baseline by 2 months. Nevertheless, conduction disturbances in two of five patients indicate that the conduction risk of PES-PFA cannot be considered negligible. The baseline LBBB in Patient 5 may reflect pre-existing conduction system vulnerability, but this small series cannot determine whether baseline conduction abnormalities, target location, or energy exposure are independent risk factors. These findings reinforce the need for careful patient selection and strict adherence to the conduction safety strategy outlined above. Larger studies with longer and more systematic rhythm monitoring are required to better define the risks of delayed atrioventricular block and permanent pacemaker implantation.

Beyond conduction-related effects, hemolysis and secondary renal injury are potential safety concerns with PFA. These risks are likely multifactorial and may be influenced by energy delivery parameters, catheter design, catheter–tissue contact, pulse burden, baseline renal function, and postprocedural hydration [27]. In an effort to mitigate these risks, we used a linear large-tip PFA catheter, maintained contact force at 10–15 g, and limited the number of ablation sites to reduce blood exposure to the electrodes and concentrate energy delivery within the septal myocardium. Serial hematologic and biochemical testing showed slight decreases in red blood cell count and hemoglobin on postprocedural day 1, accompanied by modest increases in bilirubin levels, while serum creatinine remained largely stable; the hematologic and bilirubin changes returned toward baseline by 2 months. Under the procedural strategy and energy settings used in this small series, these changes were mild and reversible and were not accompanied by clinically significant renal dysfunction. However, because hemolysis-specific markers—including lactate dehydrogenase, haptoglobin, plasma-free hemoglobin, and reticulocyte count—were not measured, the observed laboratory changes were insufficient to establish hemolysis, and subclinical hemolysis could neither be confirmed nor excluded. Although no coronary spasm or ischemic events were observed, the proximity of septal perforator branches remains an important consideration because pulsed electrical energy may provoke transient vasomotor responses [28]; careful energy titration and close periprocedural monitoring therefore remain warranted.

## 5. Limitations

This study has several limitations. It was a single-center, early exploratory study with a very small sample size, which limits statistical power and generalizability. The absence of a control group also precludes direct comparison with established SRT modalities, including surgical myectomy, alcohol septal ablation, and conventional PESA, and therefore does not allow definitive assessment of the relative efficacy or safety of PES-PFA.

Concomitant procedures may also have confounded several study outcomes. For ethical and clinical reasons, PVI was performed during the same session in two patients with paroxysmal atrial fibrillation to avoid exposing them to a separate invasive procedure. Although the final invasive transaortic pressure gradient was reassessed immediately after PES-PFA and before PVI, thereby isolating this acute hemodynamic endpoint from the subsequent PVI, the rhythm-control effect of PVI, including a potential reduction in atrial fibrillation burden, may have influenced the subsequent TTE-derived LVOT gradients and other echocardiographic parameters, as well as symptoms, exercise capacity, and quality-of-life scores. In addition, PVI in two patients and EMB in four patients may have contributed to the observed changes in cardiac injury biomarkers. Accordingly, the total procedural duration, postprocedural symptoms and functional outcomes, cardiac biomarker changes, and the overall interpretation of periprocedural adverse events cannot be attributed exclusively to PES-PFA.

Although heart rate and blood pressure were routinely monitored, the study protocol did not include time-matched recording of these variables at each invasive transaortic pressure gradient assessment; therefore, residual hemodynamic variability may have influenced the observed immediate gradient changes. Follow-up was relatively short; although early reductions in LVOT gradient, symptomatic improvement, and imaging changes were observed, the durability of benefit, the long-term remodeling response, and the long-term renal safety profile remain uncertain. CMR assessment of the ablation lesion was qualitative, and lesion volume, maximal depth, and transmural extent were not prospectively quantified. The relationship between the CMR-defined lesion and the conduction system also could not be quantified because routine CMR does not directly delineate conduction tissue and CMR–electroanatomic map coregistration was not performed. Future studies should incorporate standardized quantitative CMR analysis and integration with electroanatomic mapping.

Assessment of hemolysis was limited by the absence of hemolysis-specific markers; therefore, the transient postoperative hematologic and bilirubin changes were insufficient to establish hemolysis, and subclinical hemolysis could neither be confirmed nor excluded. Given that transient conduction disturbances occurred in two of five patients, the small sample size precludes reliable estimation of the incidence of conduction complications. Furthermore, because follow-up was limited to 2 months and only three patients underwent Holter monitoring at 2-month follow-up, asymptomatic, intermittent, or delayed conduction abnormalities cannot be fully excluded, and the long-term risk of permanent pacemaker implantation remains uncertain. Longer-term follow-up is planned at 6 and 12 months and will include 12-lead ECG, ambulatory ECG monitoring, resting and provoked TTE, CMR, and reassessment of symptoms, quality of life, and exercise capacity. These assessments are intended to detect late conduction abnormalities, characterize lesion evolution, and evaluate the durability of LVOT gradient reduction and potential recurrence of LVOTO.

In addition, the optimal energy settings, lesion extent, and procedural strategy for PES-PFA have not yet been established and will require further refinement and standardization. Because the exact pulse width, interphase delay, number of pulses per train, and pulse repetition frequency within each train were not made available to the investigators by the manufacturer, procedural reproducibility and independent assessment of dose-related safety remain limited. Finally, the mechanistic interpretations proposed in this study are hypothetical and preliminary, and will require further experimental, translational, and clinical validation.

## 6. Conclusions

This study represents the first clinical application of PFA for SRT under combined intracardiac echocardiography and three-dimensional electroanatomical mapping guidance and provides early evidence of procedural feasibility. In this small early series, PES-PFA was associated with reduced left ventricular outflow tract obstruction and improvements in symptoms and exercise capacity, with no persistent major procedure-related complications observed during short-term follow-up. However, given the very small sample size and limited follow-up, these findings should be considered hypothesis-generating only and will require confirmation in larger, preferably multicenter, studies with longer follow-up.

## Figures and Tables

**Table 1:** Baseline demographic and clinical characteristics of patients with hypertrophic cardiomyopathy treated with pulsed field ablation.

ID	Gender	Age	NYHA Functional Class	Resting (Provoked) LVOT Gradient (mmHg)	IVSd (mm)	Location of Obstruction	SAM	Pathogenic Gene Carrier Status
1	Male	53	III	51 (73)	13.7	Basal and Mid	yes	MYBPC3
2	Male	54	II	64 (88)	23.8	Basal	yes	Not detected
3	Male	59	III	76 (98)	26.5	Basal and Mid	yes	MYH7
4	Male	38	III	69 (88)	22	Basal and Mid	yes	Not detected
5	Male	67	III	41 (121)	20	Basal	yes	Not detected

LVOT = left ventricular outflow tract; IVSd = interventricular septal thickness in diastole; SAM = systolic anterior motion; NYHA = New York Heart Association.

**Table 2:** Procedural details and complications within 60 days.

Characteristic	Value
Procedural details	
LV access	
Retrograde aortic	0
Transseptal approach	5
Maximum output voltage, V	1800
Minimum output voltage, V	800
Mean number of completed septal ablation sites	29.40 ± 6.62
Mean PES-PFA energy delivery time, s	147.00 ± 33.09
Pre-PES-PFA transaortic pressure gradient, mmHg	90.80 ± 20.90
Post-PES-PFA transaortic pressure gradient, mmHg	40.20 ± 16.72
Mean total procedural duration, min	137.0 ± 25.88
Fluoroscopy time, min	29.00 ± 4.47
Mean postprocedural length of stay, days	1.60 ± 0.55
Concomitant endomyocardial biopsy	4
Concomitant pulmonary vein isolation for atrial fibrillation	2
Procedural complications	
In-hospital and 60 d mortality	0
Pericardial effusion	0
Second-degree AV block, Mobitz type II	0
AV block, complete (third-degree)	1 (transient)
Procedure-related stroke	0
Bleeding or coagulopathy	0
Vasovagal reaction	1
Right bundle branch block	0
Left bundle branch block	1 (transient)
Permanent pacemaker after the procedure	0
Ventricular septal defect	0
Ventricular tachycardia	0

Values are presented as mean ± SD or *n*. AV = atrioventricular; PES-PFA = percutaneous endocardial septal pulsed field ablation; LV = left ventricular. The post–PES-PFA transaortic pressure gradient was measured after completion of septal ablation and before pulmonary vein isolation in Patients 4 and 5. Total procedural duration included endomyocardial biopsy and pulmonary vein isolation when performed.

**Table 3:** Periprocedural and 2-month follow-up changes in echocardiographic parameters, 6 min walk distance, and KCCQ score.

Characteristic	Baseline (*n* = 5)	Immediate Postprocedure (*n* = 5)	2-Month Follow-Up (*n* = 5)
Echocardiography parameters			
Resting LVOT gradient, mmHg	60.20 ± 14.10	32.80 ± 10.92	20.60 ± 5.18
Provoked LVOT gradient, mmHg	93.60 ± 17.73	53.00 ± 11.02	27.80 ± 9.37
Maximum septal thickness, mm	21.20 ± 4.83	23.20 ± 5.20	18.98 ± 3.88
LVEF, %	58.80 ± 9.68	59.80 ± 12.19	63.40 ± 9.99
LVEDD, mm	42.90 ± 4.75	45.00 ± 3.74	50.40 ± 4.16
LVPWd, mm	13.20 ± 1.47	13.08 ± 1.56	12.06 ± 0.78
LA-ap, mm	46.40 ± 6.91	45.00 ± 7.00	44.80 ± 4.71
SAM	5	4	2
6 min Walk distance, m	272.60 ± 19.01		369.00 ± 42.87
KCCQ score, points	52.00 ± 10.56		66.40 ± 4.93

Values are presented as mean ± SD or *n*. LVOT = left ventricular outflow tract; LVEF = left ventricular ejection fraction; LVPWd = left ventricular posterior wall thickness at end-diastole; LVEDD = left ventricular end-diastolic diameter; KCCQ = Kansas City Cardiomyopathy Questionnaire; LA-ap = left atrial anteroposterior diameter; SAM = systolic anterior motion. For echocardiographic parameters, “Immediate Postprocedure” refers to assessment performed after completion of the entire index procedure, including pulmonary vein isolation when performed.

## Data Availability

The data supporting the findings of this study are available from the corresponding author upon reasonable request.

## Supplementary Materials

The following supporting information can be downloaded at https://www.mdpi.com/article/10.3390/jcm15176620/s1, Complete Inclusion and Exclusion Criteria; Supplementary Methods: Detailed Imaging Acquisition and Analysis; Supplementary Methods: Electrocardiographic and Laboratory Assessments; Supplementary Methods: Detailed Procedural Technique; Supplementary Methods: Histopathological Assessment of Endomyocardial Biopsy Specimens; Supplemental Table S1: Baseline characteristics of the 5 patients; Supplemental Table S2: Comorbidities of the 5 patients; Supplemental Table S3: Associated arrhythmias in the 5 patients; Supplemental Table S4: Preprocedural symptoms in the 5 patients; Supplemental Table S5: Patient-level preprocedural medical therapy and reasons for not undergoing surgical septal myectomy or alcohol septal ablation; Supplemental Table S6: Individual procedural details and periprocedural parameters of the 5 patients; Supplemental Table S7: Symptoms at baseline, on postprocedural day 1, and at 2-month follow-up in the 5 patients; Supplemental Table S8: Echocardiographic parameters at baseline, immediately after the procedure, and at 2-month follow-up in the 5 patients; Supplemental Table S9: Changes in NYHA functional class, 6-minute walk distance, and KCCQ score from baseline to 2-month follow-up in the 5 patients; Supplemental Table S10: Electrocardiographic parameters at baseline, immediately after the procedure, on postprocedural day 1, and at 2-month follow-up in the 5 patients; Supplemental Table S11: Complete blood count parameters at baseline, on postprocedural day 1, and at 2-month follow-up in the 5 patients; Supplemental Table S12: Biochemical parameters at baseline, on postprocedural day 1, and at 2-month follow-up in the 5 patients; Supplemental Table S13: Electrocardiographic parameters at baseline, immediately after the procedure, on postprocedural day 1, and at 2-month follow-up; Supplemental Table S14: Complete blood count and biochemical parameters at baseline, on postprocedural day 1, and at 2-month follow-up; Supplemental Figure S1: Screening, enrollment, and follow-up flow of patients evaluated for PES-PFA; Supplemental Figure S2: Fluoroscopic imaging of the endomyocardial biopsy procedure; Supplemental Figure S3: Transient left bundle branch block during PES-PFA in Patient 1; Supplemental Figure S4: Transient complete atrioventricular block during PES-PFA in Patient 5; Supplemental Figure S5: Periprocedural and 2-month changes in complete blood count parameters; Supplemental Figure S6: Periprocedural and 2-month changes in biochemical parameters; Supplementary Note: Experimental Methods and Observations for Exploratory Porcine PFA; Supplemental Video S1: Pulsed field ablation in the superior vena cava and presumed sinoatrial node regions in a porcine model; Supplemental Video S2: Complete atrioventricular block induced by pulsed field ablation at the His bundle region in a porcine model.
