# Supplementary material for: Initial Clinical Experience with Percutaneous Endocardial Septal Pulsed Field Ablation in Hypertrophic Obstructive Cardiomyopathy: A First-in-Human Case Series
Source: J Clin Med. 2026 Aug 27;15(17):6620. doi: 10.3390/jcm15176620 (PMC13566865; doi:10.3390/jcm15176620)
Supplement: Supplementary file 1 [file jcm-15-06620-s001.zip › Supplementary Materials.pdf]

# Supplementary Materials

# Initial Clinical Experience With Percutaneous Endocardial Septal Pulsed Field Ablation in Hypertrophic Obstructive Cardiomyopathy: A First-in-Human Case Series


Complete Inclusion and Exclusion Criteria .................................................. 3
Supplementary Methods: Detailed Imaging Acquisition and Analysis ......................... 5
Supplementary Methods: Electrocardiographic and Laboratory Assessments ............. 5
Supplementary Methods: Detailed Procedural Technique ............................................ 5
Supplementary Methods: Histopathological Assessment of Endomyocardial Biopsy Specimens .......................................................................................................... 7
Supplemental Table S1. Baseline characteristics of the 5 patients. ............................... 8
Supplemental Table S2. Comorbidities of the 5 patients. ............................................. 8
Supplemental Table S3. Associated arrhythmias in the 5 patients. ............................... 9
Supplemental Table S4. Preprocedural symptoms in the 5 patients. ............................. 9
Supplemental Table S5. Patient-level preprocedural medical therapy and reasons for not undergoing surgical septal myectomy or alcohol septal ablation .......................... 10
Supplemental Table S6. Individual procedural details and periprocedural parameters of the 5 patients. .......................................................................................... 11
Supplemental Table S7. Symptoms at baseline, on postprocedural day 1, and at 2-month follow-up in the 5 patients. ............................................................ 12
Supplemental Table S8. Echocardiographic parameters at baseline, immediately after the procedure, and at 2-month follow-up in the 5 patients. ....................................... 13
Supplemental Table S9. Changes in NYHA functional class, 6-minute walk distance, and KCCQ score from baseline to 2-month follow-up in the 5 patients. .................... 14
Supplemental Table S10. Electrocardiographic parameters at baseline, immediately after the procedure, on postprocedural day 1, and at 2-month follow-up in the 5 patients. ................................................................................................... 15
Supplemental Table S11. Complete blood count parameters at baseline, on postprocedural day 1, and at 2-month follow-up in the 5 patients. ............................ 16
Supplemental Table S12. Biochemical parameters at baseline, on postprocedural day 1, and at 2-month follow-up in the 5 patients. ............................................................... 17

Supplemental Table S13. Electrocardiographic parameters at baseline, immediately after the procedure, on postprocedural day 1, and at 2-month follow-up. .................. 18
Supplemental Table S14. Complete blood count and biochemical parameters at baseline, on postprocedural day 1, and at 2-month follow-up. .................................. 19
Supplemental Figure S1. Screening, enrollment, and follow-up flow of patients evaluated for PES-PFA. ................................................................................. 20
Supplemental Figure S2. Fluoroscopic imaging of the endomyocardial biopsy procedure. ................................................................................................. 21
Supplemental Figure S3. Transient left bundle branch block during PES-PFA in Patient 1. ................................................................................................. 22
Supplemental Figure S4. Transient complete atrioventricular block during PES-PFA in Patient 5. ............................................................................................ 23
Supplemental Figure S5. Periprocedural and 2-month changes in complete blood count parameters. .......................................................................................... 25
Supplemental Figure S6. Periprocedural and 2-month changes in biochemical parameters. ................................................................................................. 26
Supplementary Note: Experimental Methods and Observations for Exploratory Porcine PFA ............................................................................................ 27
Supplemental Video S1. Pulsed field ablation in the superior vena cava and presumed sinoatrial node regions in a porcine model. ............................................................. 29
Supplemental Video S2. Complete atrioventricular block induced by pulsed field ablation at the His bundle region in a porcine model. ................................................ 29

## Complete Inclusion and Exclusion Criteria

**Inclusion criteria:**

(1) Age between 18 and 80 years;

(2) LVOT gradient ≥50 mmHg at rest or upon provocation, or LVOT gradient ≥30 mmHg at rest with significant symptoms upon exertion;

(3) Interventricular septal thickness ≥13 mm;

(4) Inadequate response to beta-blockers and/or calcium channel blockers;

(5) Consent to participate in the study with a signed informed consent form.

**Exclusion criteria:**

(1) Nonobstructive HCM ;

(2) Concurrent cardiac conditions requiring surgical intervention;

(3) Severe infectious disease or malignancy;

(4) Moderate or severe hepatic impairment (Child-Pugh class B or C);

(5) Severe renal failure (estimated glomerular filtration rate <30 mL/min/1.73 $m^2$ );

(6) Echocardiography and cardiac magnetic resonance imaging suggest isolated apical hypertrophy or apical HOCM;

(7) LVOTO due to papillary muscle hypertrophy;

(8) Severe interventricular septal hypertrophy with a maximum septal thickness ≥35 mm;

(9) Presence of complete or incomplete right bundle branch block;

(10) Patients who have previously undergone ventricular septal reduction surgery;

(11) Left ventricular ejection fraction < 25%;

(12) A history of sustained ventricular tachycardia, ventricular fibrillation, or sudden cardiac arrest within the past 6 months;

(13) Patients with a confirmed thrombus in the left atrium or heart, or those with paroxysmal or persistent atrial fibrillation who have not received systemic anticoagulation treatment in the 4 weeks prior to screening;

(14) Patients with second-degree (type II) or third-degree atrioventricular block;

(15) Patients with significant congenital heart defects (e.g. atrial septal defect or severe pulmonary vein stenosis, excluding patent foramen ovale);

(16) Patients with implanted artificial heart valves;

(17) Patients with implanted cardiac pacemakers or implantable cardioverter-defibrillators;

(18) Patients with preoperative symptomatic carotid artery stenosis;

(19) Patients with untreated or uncontrolled hyperthyroidism or hypothyroidism;

(20) Patients who have experienced a myocardial infarction or undergone any cardiac intervention/open surgery within the last 3 months;

(21) Major bleeding within the past 6 months or severe coagulation dysfunction;

(22) Patients with clear contraindications to interventional surgery as determined by the investigator, and who are unable to undergo ablation surgery;

(23) Pregnant or breastfeeding women, or women planning pregnancy during the study period;

(24) Patients who have participated in other clinical trials involving drugs or medical devices within the past 3 months;

(25) Any other conditions deemed unsuitable for participation by the investigators.

**Supplementary Methods: Detailed Imaging Acquisition and Analysis**

TTE was performed using an EPIQ 7C system (Philips Medical Systems, USA) with S5-1 and X5-1 transducers (1.0 – 5.0 MHz). Images were stored in DICOM format for offline analysis. CMR was performed on an Ingenia 1.5-T scanner (Philips Medical Systems, USA) with a 32-channel body coil. Respiratory and electrocardiographic gating were used. Gadobutrol (0.1 mmol/kg; Bayer Healthcare, Germany) was administered intravenously, and late gadolinium enhancement imaging was used to assess septal enhancement.

**Supplementary Methods: Electrocardiographic and Laboratory Assessments**

All patients underwent 24-hour ambulatory electrocardiogram (ECG) monitoring before the procedure using the Cardio Trak CT-082 system (Hangzhou Baihui, China) to characterize baseline rhythm and identify additional arrhythmias or conduction abnormalities. Standard 12-lead ECGs were obtained at baseline, immediately after completion of the index procedure, on postprocedural day 1, and at 2-month follow-up using the MAC 1200ST system (GE Healthcare) to assess changes in cardiac rhythm and conduction. PR intervals were assessed on ECG tracings recorded during sinus rhythm. All patients underwent continuous telemetry monitoring for 48 hours after the procedure. At 2-month follow-up, Patients 1, 4, and 5 underwent additional 24-hour Holter monitoring. Holter monitoring was performed in Patients 1 and 5 because of intraprocedural conduction disturbances and in Patients 4 and 5 because they had undergone pulmonary vein isolation.

Peripheral venous blood samples were obtained at baseline, on postprocedural day 1, and at 2-month follow-up. Laboratory testing included complete blood count, liver and renal function tests, and measurement of cardiac injury biomarkers. These assessments were performed to evaluate periprocedural hematologic changes, myocardial injury, and hepatic or renal dysfunction.

**Supplementary Methods: Detailed Procedural Technique**

Written informed consent for the procedure was obtained from all patients before the

procedure. Patients were placed supine with continuous ECG and oxygen saturation monitoring and intermittent noninvasive blood pressure monitoring. The groin region and right upper extremity were prepared and draped in a sterile fashion, and local anesthesia was achieved with 1% lidocaine.

The left femoral vein and right radial artery were cannulated. A temporary pacing lead was advanced into the right ventricle via the left femoral vein for backup pacing, and a decapolar catheter was positioned in the coronary sinus. A 7-F angiographic catheter was introduced through the right radial artery and advanced retrogradely to the left ventricular apex for invasive pressure assessment. The pressure-recording system was calibrated before each assessment. Pressures were measured sequentially in the left ventricle and above the aortic valve using the same pullback protocol, and the transaortic pressure gradient was calculated from multiple consecutive stable beats. Measurements during premature ventricular contractions, ventricular tachycardia, or postextrasystolic beats were excluded.

The right femoral vein was then cannulated, and an SL1 long sheath followed by an Agilis steerable sheath was introduced. In Patients 1–4, right ventricular and pulmonary artery angiography was performed to identify suitable sites for right ventricular septal endomyocardial biopsy (Supplemental Figure S2A). Biopsy specimens were obtained with biopsy forceps from the basal, mid, and apical portions of the interventricular septum (Supplemental Figures S2B to S2D). The biopsy procedure and its potential risks were discussed with the patients during the preprocedural informed-consent process, and written informed consent was obtained. Patient 5 did not undergo biopsy because pre-existing left bundle branch block raised concern that right bundle branch injury during right ventricular septal biopsy could precipitate complete atrioventricular block. After tissue sampling, transseptal puncture was performed to advance the sheath into the left atrium, and a catheter was introduced into the left ventricle for LVOT and aortic angiography. The angiographic catheter was then exchanged for a PFA catheter with pressure-monitoring capability (AForcePlus™, Access Point Technologies Medical, China).

After removal of the SL1 sheath, an 11-F sheath was inserted, through which a

10-F ICE catheter (SOUNDSTAR™, Biosense Webster, USA) was advanced across the tricuspid valve into the mid right ventricle, with its tip oriented toward the right ventricular apex. After release of the anterior deflection used to facilitate passage into the right ventricle, the catheter was gradually rotated clockwise and leftward deflection was applied to bring the left ventricle into the imaging sector and obtain a left ventricular long-axis view. Small adjustments in left/right deflection were then used to sweep the imaging plane across the basal and mid interventricular septum, allowing visualization of the hypertrophied obstructive septal segment and its relationship to the LVOT and thereby guiding target definition.

Throughout the procedure, ICE was also used to assess catheter-septal contact and to monitor for valvular injury and pericardial effusion. A bipolar electrode catheter was maintained at the right ventricular apex for temporary pacing support when needed. In the event of postprocedural hemodynamic instability, intra-aortic balloon pump support could be initiated. At the end of the procedure, all catheters and sheaths were removed, and hemostasis was achieved by local compression followed by pressure dressing.

**Supplementary Methods: Histopathological Assessment of Endomyocardial Biopsy Specimens**

Endomyocardial biopsy specimens from Patients 1–4 underwent the same histopathological assessment. The specimens were evaluated using hematoxylin and eosin, Masson trichrome, phosphotungstic acid hematoxylin, elastic fiber, periodic acid–Schiff, and Congo red staining. Congo red-stained sections were additionally examined under polarized light. Immunohistochemical staining for CD3 and CD4 was also performed.

**Supplemental Table S1. Baseline characteristics of the 5 patients.**

| Patient | Basic Information | | | | | | | |
|---|---|---|---|---|---|---|---|---|
| | Age | Gender | Weight (kg) | Height (m) | BMI | Smoking history | Drinking History | Risk of SCD at 5 years, % |
| 1 | 53 | Male | 77.5 | 1.6 | 30.3 | 1 | 0 | 1.6 |
| 2 | 54 | Male | 77 | 1.75 | 25.1 | 0 | 0 | 2.68 |
| 3 | 59 | Male | 80 | 1.73 | 26.7 | 0 | 1 | 3.25 |
| 4 | 38 | Male | 65 | 1.7 | 22.5 | 1 | 1 | 5.21 |
| 5 | 67 | Male | 80 | 1.68 | 28.3 | 0 | 0 | 1.93 |

BMI = body mass index and was calculated as weight (kg) divided by height ($m^2$); SCD = sudden cardiac death. Five-year SCD risk was calculated according to the 2014 European Society of Cardiology guidelines for hypertrophic cardiomyopathy. For smoking history and drinking history, 1 indicates presence and 0 indicates absence.

**Supplemental Table S2. Comorbidities of the 5 patients.**

| Patient | Comorbidities | | | | | | |
|---|---|---|---|---|---|---|---|
| | Heart Failure | NYHA Functional Classification | Coronary Heart Disease | Hypertension | Diabetes Mellitus | Hyperlipidemia | Chronic Kidney Disease |
| 1 | 0 | III | 1 | 1 | 0 | 1 | 0 |
| 2 | 0 | II | 1 | 0 | 0 | 0 | 0 |
| 3 | 0 | III | 0 | 1 | 0 | 0 | 0 |
| 4 | 0 | III | 0 | 0 | 0 | 0 | 0 |
| 5 | 1 | III | 0 | 0 | 0 | 0 | 0 |

For heart failure, coronary heart disease, hypertension, diabetes mellitus, hyperlipidemia, and chronic kidney disease, 1 indicates presence and 0 indicates absence. NYHA = New York Heart Association.

**Supplemental Table S3. Associated arrhythmias in the 5 patients.**

| Patient | Associated Arrhythmia | | | | | | |
|---|---|---|---|---|---|---|---|
| | Premature atrial contractions | Atrial Tachycardia | Atrial Flutter | Atrial Fibrillation | Supraventricular Tachycardia | Premature ventricular contractions | Ventricular Tachycardia |
| 1 | 1 | 0 | 0 | 0 | 0 | 1 | 0 |
| 2 | 1 | 0 | 0 | 0 | 0 | 1 | 0 |
| 3 | 1 | 0 | 0 | 0 | 0 | 1 | 0 |
| 4 | 1 | 1 | 0 | 1 | 0 | 0 | 0 |
| 5 | 0 | 0 | 0 | 1 | 0 | 0 | 0 |

For associated arrhythmias, 1 indicates presence and 0 indicates absence.

All patients underwent preprocedural 24-hour ambulatory ECG monitoring to assess malignant arrhythmias. Occasional premature atrial and/or ventricular contractions were detected in 4 patients. Two patients had atrial fibrillation, including 1 with concomitant atrial tachycardia. On humanitarian grounds and according to patient preference, both patients with atrial fibrillation underwent pulmonary vein isolation during the septal ablation procedure. Patients with benign arrhythmias were treated medically and followed clinically.

**Supplemental Table S4. Preprocedural symptoms in the 5 patients.**

| Patient | Symptom | | | | | |
|---|---|---|---|---|---|---|
| | Dyspnea | Fatigue | Chest pain | Dizziness | Palpitations | Syncope or Pre-syncope |
| 1 | 0 | 1 | 1 | 0 | 0 | 0 |
| 2 | 0 | 1 | 1 | 0 | 1 | 0 |
| 3 | 0 | 1 | 1 | 0 | 0 | 0 |
| 4 | 0 | 1 | 1 | 0 | 1 | 0 |
| 5 | 0 | 1 | 1 | 1 | 1 | 0 |

For symptoms, 1 indicates presence and 0 indicates absence.

Symptoms were recorded at the time of admission. To comprehensively capture symptom information and minimize bias related to physician-directed questioning, symptom data were collected using a combination of questionnaires and medical records. The same physician collected symptom data for all patients using standardized clinical interviews and was not involved in the PES-PFA procedure.

**Supplemental Table S5. Patient-level preprocedural medical therapy and reasons for not undergoing surgical septal myectomy or alcohol septal ablation**

| Patient | HOCM-directed therapy | Response/limitation to medical therapy | Other concomitant cardiovascular medications | Reason for not undergoing myectomy | Reason for not undergoing ASA |
|---|---|---|---|---|---|
| 1 | Metoprolol 100 mg BID → diltiazem 120 mg TID [a] | Persistent symptoms/LVOTO | Clopidogrel 75 mg QD | Patient declined [b] | No suitable septal perforator identified |
| 2 | Metoprolol 100 mg BID → diltiazem 120 mg TID [a] | Persistent symptoms/LVOTO | Clopidogrel 75 mg QD | Patient declined [b] | No suitable septal perforator identified |
| 3 | Bisoprolol 5 mg BID | Symptomatic hypotension with CCB [c] | None | Patient declined [b] | Severe target septal perforator tortuosity [d] |
| 4 | Metoprolol 100 mg BID → diltiazem 120 mg TID [a] | Persistent symptoms/LVOTO | Edoxaban 60 mg QD | Elevated perioperative risk; Patient declined [e] | No suitable septal perforator identified |
| 5 | Metoprolol 50 mg BID | β-blocker uptitration limited; CCB avoided [f] | Sacubitril/valsartan 50 mg BID; Spironolactone 20 mg QD; Empagliflozin 10 mg QD; Vericiguat 2.5 mg QD; Rivaroxaban 20 mg QD | High conduction-block risk [g] | High conduction-block risk |

[a] Metoprolol and diltiazem were administered sequentially rather than concomitantly in Patients 1, 2, and 4. Despite sequential trials of high-dose therapy, clinically significant symptoms and LVOTO persisted.

[b] Patients 1 and 2 declined surgical septal myectomy after counseling because of concerns regarding the invasiveness and surgical trauma of open-heart surgery and a preference for a less invasive treatment. Patient 3 declined surgical septal myectomy because of concerns regarding surgical trauma and an unwillingness to accept the potential risk of conduction-system injury.

[c] In Patient 3, addition of a nondihydropyridine calcium channel blocker resulted in symptomatic hypotension and could not be continued.

[d] In Patient 3, severe tortuosity of the target septal perforator precluded stable balloon positioning and occlusion.

[e] Patient 4 had concomitant cerebrovascular disease associated with increased perioperative neurologic and anesthetic risk and declined surgical septal myectomy after counseling.

[f] In Patient 5, further uptitration of metoprolol caused symptomatic hypotension and bradycardia. Nondihydropyridine calcium channel blockers were avoided because of

HFrEF.

[g] Following comprehensive multidisciplinary evaluation, Patient 5 was considered unsuitable for either surgical septal myectomy or ASA because pre-existing LBBB was associated with a high risk of clinically significant conduction block, while concomitant HFrEF further increased the overall procedural risk.

ASA indicates alcohol septal ablation; AV, atrioventricular; CCB, calcium channel blocker; HOCM, hypertrophic obstructive cardiomyopathy; HFrEF, heart failure with reduced ejection fraction; LBBB, left bundle branch block; LVOTO, left ventricular outflow tract obstruction; QD, once daily; BID, twice daily; and TID, three times daily.

**Supplemental Table S6. Individual procedural details and periprocedural parameters of the 5 patients.**

| Patient | Procedural details | | | | |
|---|---|---|---|---|---|
| | LV access | Number of completed septal ablation sites | PES-PFA energy-delivery time (s) | Concomitant pulmonary vein isolation | Concomitant endomyocardial biopsy |
| 1 | Transseptal approach | 28 | 140 | 0 | 1 |
| 2 | Transseptal approach | 27 | 135 | 0 | 1 |
| 3 | Transseptal approach | 40 | 200 | 0 | 1 |
| 4 | Transseptal approach | 30 | 150 | 1 | 1 |
| 5 | Transseptal approach | 22 | 110 | 1 | 0 |
| Patient | Procedural details | | | | |
| | Pre-PES-PFA transaortic pressure gradient (mmHg) | Post-PES-PFA transaortic pressure gradient (mmHg) | Fluoroscopy time (min) | Total procedural duration (min) | Postprocedural length of stay (days) |
| 1 | 59 | 32 | 23 | 130 | 2 |
| 2 | 100 | 51 | 29 | 115 | 2 |
| 3 | 98 | 19 | 31 | 140 | 1 |
| 4 | 83 | 62 | 27 | 180 | 1 |
| 5 | 114 | 37 | 35 | 120 | 2 |
| Patient | Procedural details | | | | |
| | Heart rate before/after PES-PFA, beats/min | | Noninvasive blood pressure before/after PES-PFA, mmHg | | |
| 1 | 75 | 76 | 100/52 | 127/89 | |
| 2 | 68 | 72 | 121/87 | 115/86 | |
| 3 | 88 | 95 | 127/76 | 123/91 | |
| 4 | 65 | 73 | 128/76 | 139/67 | |
| 5 | 61 | 65 | 105/63 | 117/78 | |

LV = left ventricular; PES-PFA = percutaneous endocardial septal pulsed field ablation. For concomitant pulmonary vein isolation and concomitant endomyocardial biopsy, 1 indicates performed and 0 indicates not performed. Transaortic pressure

gradient was measured immediately before and after PES-PFA. In Patients 4 and 5, the post–PES–PFA measurement was completed before subsequent pulmonary vein isolation. Total procedural duration included concomitant endomyocardial biopsy and pulmonary vein isolation when performed. Heart rate and noninvasive blood pressure values were retrospectively extracted from routine intraoperative monitoring records and represent the last available readings before PES-PFA and the first available readings after completion of PES-PFA. These measurements were obtained at 15-minute intervals and were not time-matched to the invasive transaortic pressure gradient assessments.

**Supplemental Table S7. Symptoms at baseline, on postprocedural day 1, and at 2-month follow-up in the 5 patients.**

| Patient | Symptom | | | | | | | | | | | |
|---|---|---|---|---|---|---|---|---|---|---|---|---|
| | Dyspnea | | Fatigue | | Chest pain | | Dizziness | | Palpitations | | Syncope or Pre-syncope | |
| | Baseline | POD 1 | Baseline | POD 1 | Baseline | POD 1 | Baseline | POD 1 | Baseline | POD 1 | Baseline | POD 1 |
| 1 | 0 | 0 | 1 | 1 | 1 | 1 | 0 | 0 | 0 | 0 | 0 | 0 |
| 2 | 0 | 0 | 1 | 0 | 1 | 0 | 0 | 0 | 1 | 1 | 0 | 0 |
| 3 | 0 | 0 | 1 | 1 | 1 | 1 | 0 | 0 | 0 | 0 | 0 | 0 |
| 4 | 0 | 0 | 1 | 0 | 1 | 0 | 0 | 0 | 1 | 0 | 0 | 0 |
| 5 | 0 | 0 | 1 | 0 | 1 | 0 | 1 | 0 | 1 | 0 | 0 | 0 |
| Patient | Symptom | | | | | | | | | | | |
| | Dyspnea | | Fatigue | | Chest pain | | Dizziness | | Palpitations | | Syncope or Pre-syncope | |
| | Baseline | 2-mo FU | Baseline | 2-mo FU | Baseline | 2-mo FU | Baseline | 2-mo FU | Baseline | 2-mo FU | Baseline | 2-mo FU |
| 1 | 0 | 0 | 1 | 0 | 1 | 0 | 0 | 0 | 0 | 0 | 0 | 0 |
| 2 | 0 | 0 | 1 | 0 | 1 | 0 | 0 | 0 | 1 | 1 | 0 | 0 |
| 3 | 0 | 0 | 1 | 1 | 1 | 1 | 0 | 0 | 0 | 0 | 0 | 0 |
| 4 | 0 | 0 | 1 | 0 | 1 | 0 | 0 | 0 | 1 | 0 | 0 | 0 |
| 5 | 0 | 0 | 1 | 0 | 1 | 0 | 1 | 0 | 1 | 0 | 0 | 0 |

BL, baseline; POD 1, postprocedural day 1; 2-mo FU, 2-month follow-up.

For each symptom, 1 indicates presence and 0 indicates absence. Symptoms were assessed at baseline, on postprocedural day 1, and at 2-month follow-up using a combination of questionnaires and medical records. The same physician documented

symptom data before the procedure and during follow-up using standardized clinical interviews. This physician was not involved in the procedural performance of PES-PFA, and data analysis was performed by a different physician.

**Supplemental Table S8. Echocardiographic parameters at baseline, immediately after the procedure, and at 2-month follow-up in the 5 patients.**

| Patient | Echocardiographic parameters | | | | | | | | |
|---|---|---|---|---|---|---|---|---|---|
| | Resting LVOT gradient (mmHg) | | | Provoked LVOT gradient (mmHg) | | | Maximum septal thickness (mm) | | |
| | Baseline | Immediate Postprocedure | 2-Month Follow-up | Baseline | Immediate Postprocedure | 2-Month Follow-up | Baseline | Immediate Postprocedure | 2-Month Follow-up |
| 1 | 51 | 35 | 27 | 73 | 47 | 39 | 13.7 | 14.7 | 13 |
| 2 | 64 | 31 | 13 | 88 | 65 | 16 | 23.8 | 25.6 | 20 |
| 3 | 76 | 42 | 19 | 98 | 64 | 22 | 26.5 | 27.9 | 23.5 |
| 4 | 69 | 41 | 23 | 88 | 49 | 35 | 22 | 25.8 | 20.4 |
| 5 | 41 | 15 | 21 | 121 | 40 | 27 | 20 | 22 | 18 |

| Patient | Echocardiographic parameters | | | | | | | | |
|---|---|---|---|---|---|---|---|---|---|
| | LVEF (%) | | | LVEDD (mm) | | | LVPWd (mm) | | |
| | Baseline | Immediate Postprocedure | 2-Month Follow-up | Baseline | Immediate Postprocedure | 2-Month Follow-up | Baseline | Immediate Postprocedure | 2-Month Follow-up |
| 1 | 57 | 65 | 65 | 42 | 47 | 48 | 11.3 | 11.1 | 11.1 |
| 2 | 70 | 68 | 70 | 50 | 47 | 52 | 13.5 | 11.9 | 11.7 |
| 3 | 59 | 59 | 66 | 38.5 | 41 | 48 | 15 | 15 | 12.7 |
| 4 | 64 | 68 | 70 | 39 | 41 | 47 | 14 | 13.7 | 13 |
| 5 | 44 | 39 | 46 | 45 | 49 | 57 | 12.2 | 13.7 | 11.8 |

| Patient | Echocardiographic parameters | | | | | |
|---|---|---|---|---|---|---|
| | LA-ap (mm) | | | SAM | | |
| | Baseline | Immediate Postprocedure | 2-Month Follow-up | Baseline | Immediate Postprocedure | 2-Month Follow-up |
| 1 | 40 | 40 | 41 | 1 | 1 | 1 |
| 2 | 41 | 40 | 41 | 1 | 1 | 0 |
| 3 | 49 | 48 | 52 | 1 | 1 | 0 |
| 4 | 57 | 56 | 47 | 1 | 1 | 1 |
| 5 | 45 | 41 | 43 | 1 | 0 | 0 |

| Patient | Echocardiographic parameters | | |
|---|---|---|---|
| | Mitral regurgitation severity | | |
| | Baseline | Immediate Postprocedure | 2-Month Follow-up |
| 1 | Moderate | Mild-to-moderate | Mild-to-moderate |
| 2 | Moderate | Mild | Mild |
| 3 | Moderate | Moderate | Mild |
| 4 | Severe | Moderate-to-severe | Moderate |

| | | | |
|---|---|---|---|
| 5 | Moderate | Mild | Mild |

LVOT = left ventricular outflow tract; LVEF = left ventricular ejection fraction; LVEDD = left ventricular end-diastolic diameter; LVPWd = left ventricular posterior wall thickness at end-diastole; LA-ap = left atrial anteroposterior diameter; SAM = systolic anterior motion. For SAM, 1 indicates presence and 0 indicates absence. "Immediate Postprocedure" refers to transthoracic echocardiographic assessment performed after completion of the entire index procedure, including pulmonary vein isolation when performed. Mitral regurgitation severity was graded using an integrated echocardiographic assessment.

All 5 patients underwent transthoracic echocardiography at baseline, immediately after completion of the index procedure, and at 2-month follow-up to assess cardiac structure and function. To minimize measurement variability, all examinations were performed using the same echocardiography system by a single experienced echocardiographer. Relevant parameters were measured repeatedly and averaged for analysis.

**Supplemental Table S9. Changes in NYHA functional class, 6-minute walk distance, and KCCQ score from baseline to 2-month follow-up in the 5 patients.**

| Patient | NYHA Functional Classification | | 6-min Walk distance (m) | | KCCQ score (points) | |
|---|---|---|---|---|---|---|
| | Baseline | 2-Month Follow-up | Baseline | 2-Month Follow-up | Baseline | 2-Month Follow-up |
| 1 | III | III | 245 | 372 | 45 | 67 |
| 2 | II | II | 270 | 323 | 69 | 70 |
| 3 | III | II | 268 | 348 | 54 | 72 |
| 4 | III | II | 295 | 438 | 42 | 63 |
| 5 | III | II | 285 | 364 | 50 | 60 |

KCCQ = Kansas City Cardiomyopathy Questionnaire; NYHA = New York Heart Association.

Exercise tolerance and health status were assessed using NYHA functional class, 6-minute walk distance, and KCCQ score. All 3 assessments were performed at baseline and at 2-month follow-up. NYHA functional class was determined using a combination of standardized questionnaires and clinical interviews. The same

physician, who was not involved in the procedural performance of PES-PFA, performed the NYHA assessments at both time points. The 6-minute walk test was performed under conditions ensuring patient safety and clinical suitability for exertion and was supervised by designated personnel. All 5 patients completed the test. The KCCQ was completed independently by each patient at both time points.

**Supplemental Table S10. Electrocardiographic parameters at baseline, immediately after the procedure, on postprocedural day 1, and at 2-month follow-up in the 5 patients.**

| Patient | Electrocardiographic parameters | | | | | | | |
|---|---|---|---|---|---|---|---|---|
| | Heart rate (beats/min) | | | | PR interval (ms) | | | |
| | Baseline | Immediate Postprocedure | Postprocedural Day 1 | 2-Month Follow-up | Baseline | Immediate Postprocedure | Postprocedural Day 1 | 2-Month Follow-up |
| 1 | 58 | 72 | 75 | 70 | 166 | 178 | 176 | 164 |
| 2 | 72 | 85 | 68 | 73 | 168 | 168 | 170 | 164 |
| 3 | 82 | 95 | 72 | 80 | 156 | 168 | 162 | 186 |
| 4 | 65 | 89 | 55 | 73 | 172 | 190 | 178 | 170 |
| 5 | 62 | 62 | 66 | 59 | 192 | 200 | 186 | 188 |

| Patient | Electrocardiographic parameters | | | | | | | |
|---|---|---|---|---|---|---|---|---|
| | QRS duration (ms) | | | | QTc interval (ms) | | | |
| | Baseline | Immediate Postprocedure | Postprocedural Day 1 | 2-Month Follow-up | Baseline | Immediate Postprocedure | Postprocedural Day 1 | 2-Month Follow-up |
| 1 | 94 | 96 | 98 | 98 | 455 | 438 | 457 | 442 |
| 2 | 104 | 114 | 114 | 104 | 448 | 454 | 423 | 445 |
| 3 | 104 | 102 | 104 | 100 | 441 | 459 | 440 | 440 |
| 4 | 106 | 116 | 116 | 106 | 409 | 464 | 420 | 449 |
| 5 | 192 | 194 | 204 | 194 | 523 | 531 | 551 | 536 |

QTc = corrected QT interval. PR intervals were measured from ECGs recorded during sinus rhythm.

To monitor ECG changes and assess conduction abnormalities, standard 12-lead ECGs were obtained in all 5 patients at baseline, immediately after completion of the index procedure, on postprocedural day 1, and at 2-month follow-up. All ECG examinations were performed according to a standardized protocol by the same

electrophysiologist, who was not involved in the procedural performance of PES-PFA. ECG data were stored in the electrocardiography workstation, and the relevant parameters were automatically measured and uniformly exported for analysis. Baseline ECG assessment was used to characterize preprocedural conduction status, whereas follow-up measurements were compared with baseline values to assess postprocedural changes in cardiac conduction.

**Supplemental Table S11. Complete blood count parameters at baseline, on postprocedural day 1, and at 2-month follow-up in the 5 patients.**

| Patient | Complete blood count parameters | | | | | | | | |
|---|---|---|---|---|---|---|---|---|---|
| | RBC ($\times10^{12}$/L) | | | HGB (g/L) | | | WBC ($\times10^{9}$/L) | | |
| | Baseline | Postprocedural Day 1 | 2-Month Follow-up | Baseline | Postprocedural Day 1 | 2-Month Follow-up | Baseline | Postprocedural Day 1 | 2-Month Follow-up |
| 1 | 4.69 | 4.64 | 4.57 | 144 | 143 | 142 | 6.25 | 7.6 | 6.95 |
| 2 | 4.65 | 4.78 | 5.39 | 139 | 137 | 155 | 4.41 | 6.32 | 4.59 |
| 3 | 5.77 | 5.4 | 5.81 | 173 | 161 | 172 | 7.98 | 8.89 | 7.31 |
| 4 | 4.45 | 4.45 | 4.98 | 150 | 152 | 153 | 7 | 7.79 | 7.61 |
| 5 | 4.34 | 4.25 | 4.13 | 131 | 129 | 129 | 10.02 | 12.27 | 7.19 |
| Patient | Complete blood count parameters | | | | | | | | |
| | Neutrophil count ($\times10^{9}$/L) | | | Neutrophil percentage (%) | | | PLT ($\times10^{9}$/L) | | |
| | Baseline | Postprocedural Day 1 | 2-Month Follow-up | Baseline | Postprocedural Day 1 | 2-Month Follow-up | Baseline | Postprocedural Day 1 | 2-Month Follow-up |
| 1 | 3.93 | 5.2 | 3.88 | 62.9 | 68.4 | 55.8 | 167 | 176 | 199 |
| 2 | 2.53 | 4.04 | 2.63 | 61 | 63.9 | 57.3 | 122 | 109 | 127 |
| 3 | 5.49 | 6.88 | 4.62 | 68.8 | 77.4 | 63.2 | 214 | 193 | 196 |
| 4 | 3.58 | 5.77 | 4.55 | 51.1 | 74 | 59.8 | 204 | 165 | 189 |
| 5 | 7.73 | 11.6 | 4.8 | 77.1 | 90.9 | 66.8 | 274 | 277 | 251 |

RBC = red blood cell count; HGB = hemoglobin; WBC = white blood cell count; PLT = platelet count.

Complete blood count parameters were measured in all 5 patients at baseline, on postprocedural day 1, and at 2-month follow-up. Testing was performed using an automated hematology analyzer (CAL 8000, Mindray Medical), and all results were uniformly exported through the laboratory information system of the First Affiliated Hospital of Harbin Medical University.

**Supplemental Table S12. Biochemical parameters at baseline, on postprocedural day 1, and at 2-month follow-up in the 5 patients.**

| Patient | Biochemical parameters | | | | | | | | |
|---|---|---|---|---|---|---|---|---|---|
| | cTnI (ng/L) | | | CK-MB (ng/mL) | | | TBIL (μmol/L) | | |
| | Baseline | Postprocedural Day 1 | 2-Month Follow-up | Baseline | Postprocedural Day 1 | 2-Month Follow-up | Baseline | Postprocedural Day 1 | 2-Month Follow-up |
| 1 | 189.5 | 2791.8 | 36.8 | 4.1 | 23 | 4.3 | 9.1 | 19.3 | 12.8 |
| 2 | 137.1 | 7222.7 | 46.2 | 3.5 | 16 | 3.8 | 15.22 | 23.6 | 7.9 |
| 3 | 25.5 | 2015 | 14.7 | 1.4 | 13.7 | 1.2 | 34 | 42 | 22 |
| 4 | 121.7 | 4633 | 12.9 | 4.5 | 10.5 | 3.7 | 11.67 | 18.4 | 6.4 |
| 5 | 373.3 | 3529.9 | 372 | 1.9 | 5.64 | 3.5 | 10.8 | 13.35 | 11.29 |
| Patient | Biochemical parameters | | | | | | | | |
| | DBIL (μmol/L) | | | IBIL (μmol/L) | | | Cr (μmol/L) | | |
| | Baseline | Postprocedural Day 1 | 2-Month Follow-up | Baseline | Postprocedural Day 1 | 2-Month Follow-up | Baseline | Postprocedural Day 1 | 2-Month Follow-up |
| 1 | 1.5 | 3.2 | 2.3 | 7.6 | 16.1 | 10.5 | 55 | 63 | 68 |
| 2 | 5.27 | 7.4 | 1.8 | 9.95 | 16.2 | 6.1 | 81.7 | 80 | 94 |
| 3 | 6.6 | 8.4 | 4.4 | 27.4 | 33.6 | 17.6 | 73.8 | 71.7 | 79.6 |
| 4 | 3.31 | 5.7 | 1.1 | 8.36 | 12.7 | 5.3 | 80.7 | 73.5 | 59.7 |
| 5 | 2.2 | 4.26 | 3.49 | 8.6 | 9.29 | 7.8 | 81 | 85.1 | 88.2 |

cTnI = cardiac troponin I; CK-MB = creatine kinase-MB isoenzyme; TBIL = total bilirubin; DBIL = direct bilirubin; IBIL = indirect bilirubin; Cr = creatinine.

To assess periprocedural myocardial injury and biochemical changes, laboratory tests were performed at baseline, on postprocedural day 1, and at 2-month follow-up. Testing was performed using a fully automated biochemical analyzer (BS-2000M, Mindray Medical) and an immunoassay analyzer (Alinity, Abbott Laboratories). All results were uniformly exported through the laboratory information system of the First Affiliated Hospital of Harbin Medical University.

**Supplemental Table S13. Electrocardiographic parameters at baseline, immediately after the procedure, on postprocedural day 1, and at 2-month follow-up.**

| Characteristic | Baseline (n=5) | Immediate Postprocedure (n=5) | Postprocedural Day 1 (n=5) | 2-Month Follow-up (n=5) |
|---|---|---|---|---|
| Heart rate, beats/min | 67.80 ± 9.45 | 80.60 ± 13.39 | 67.20 ± 7.66 | 71.00 ± 7.65 |
| PR interval, ms | 170.80 ± 13.24 | 180.80 ± 14.04 | 174.40 ± 8.99 | 174.40 ± 11.78 |
| QRS duration, ms | 120.00 ± 40.52 | 124.40 ± 39.78 | 127.20 ± 43.56 | 120.40 ± 41.26 |
| QTc interval, ms | 455.20 ± 41.79 | 469.20 ± 35.90 | 458.20 ± 53.95 | 462.40 ± 41.28 |
| Low QRS voltage | 0 | 0 | 0 | 0 |
| Right bundle branch block | 0 | 0 | 0 | 0 |
| Left bundle branch block | 1 | 1 | 1 | 1 |
| Atrioventricular block (all types) | 0 | 0 | 0 | 0 |
| Ventricular tachycardia (all types) | 0 | 0 | 0 | 0 |
| Premature ventricular contractions | 0 | 0 | 0 | 0 |

Values are presented as mean ± SD or n. QTc = corrected QT interval. PR intervals were assessed on electrocardiograms recorded during sinus rhythm. Categorical rhythm and conduction findings in this table were based on standard 12-lead ECGs obtained at the scheduled assessment time points and do not include transient intraprocedural events or arrhythmias detected only on ambulatory monitoring.

**Supplemental Table S14. Complete blood count and biochemical parameters at baseline, on postprocedural day 1, and at 2-month follow-up.**

| Parameter | Baseline (n=5) | Postprocedural Day 1 (n=5) | 2-Month Follow-up (n=5) |
|---|---|---|---|
| Complete blood count parameters | | | |
| RBC ($\times 10^{12}$/L) | 4.78 ± 0.57 | 4.70 ± 0.44 | 4.98 ± 0.66 |
| HGB (g/L) | 147.40 ± 15.92 | 144.40 ± 12.52 | 150.20 ± 15.99 |
| WBC ($\times 10^{9}$/L) | 7.13 ± 2.08 | 8.57 ± 2.26 | 6.73 ± 1.22 |
| Neutrophil count ($\times 10^{9}$/L) | 4.65 ± 2.02 | 6.70 ± 2.93 | 4.10 ± 0.89 |
| Neutrophil percentage (%) | 64.18 ± 9.63 | 74.92 ± 10.32 | 60.58 ± 4.47 |
| PLT ($\times 10^{9}$/L) | 196.20 ± 56.54 | 184.00 ± 60.79 | 192.40 ± 44.10 |
| Biochemical parameters | | | |
| cTnI (ng/L) | 169.40 ± 128.50 | 4038.00 ± 2025.00 | 96.52 ± 154.70 |
| CK-MB (ng/mL) | 3.08 ± 1.37 | 13.77 ± 6.46 | 3.30 ± 1.21 |
| TBIL (μmol/L) | 16.16 ± 10.22 | 23.33 ± 11.05 | 12.08 ± 6.11 |
| DBIL (μmol/L) | 3.78 ± 2.13 | 5.79 ± 2.15 | 2.62 ± 1.32 |
| IBIL (μmol/L) | 12.38 ± 8.44 | 17.58 ± 9.40 | 9.46 ± 4.97 |
| Cr (μmol/L) | 74.44 ± 11.33 | 74.66 ± 8.42 | 77.90 ± 14.12 |

Values are presented as mean ± SD. RBC = red blood cell count; HGB = hemoglobin; WBC = white blood cell count; PLT = platelet count; cTnI = cardiac troponin I; CK-MB = creatine kinase-MB isoenzyme; TBIL = total bilirubin; DBIL = direct bilirubin; IBIL = indirect bilirubin; Cr = creatinine.

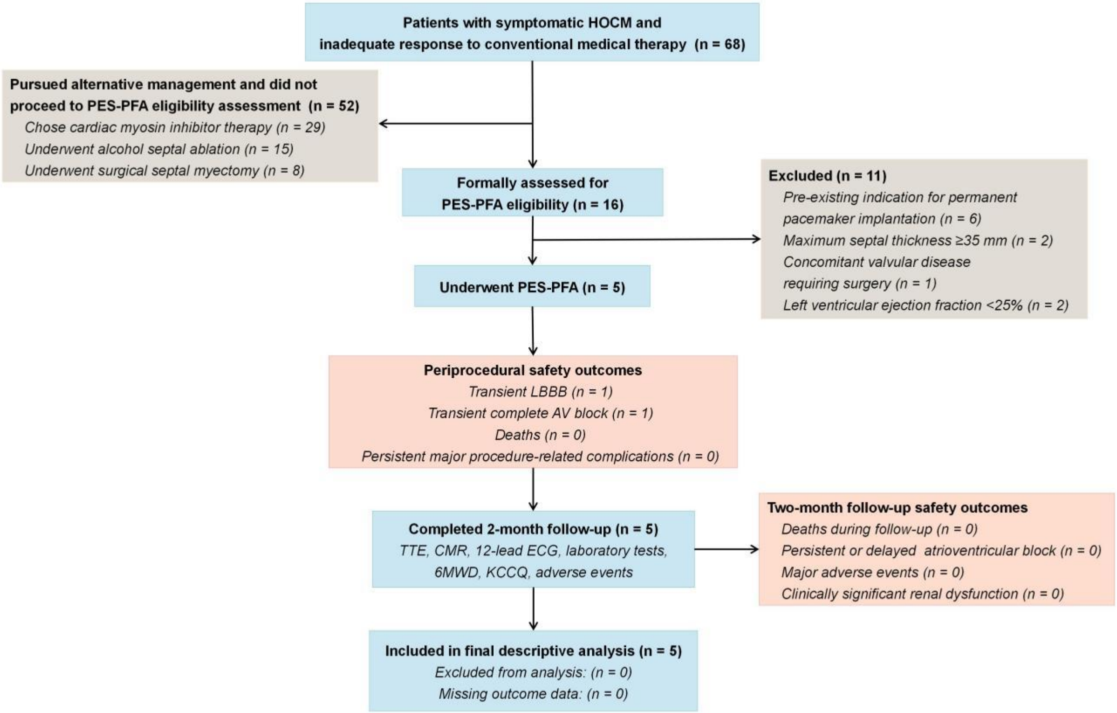


**Supplemental Figure S1. Screening, enrollment, and follow-up flow of patients evaluated for PES-PFA.**

AV indicates atrioventricular; CMR, cardiac magnetic resonance; ECG, electrocardiography; HOCM, hypertrophic obstructive cardiomyopathy; KCCQ, Kansas City Cardiomyopathy Questionnaire; LBBB, left bundle branch block; PES-PFA, percutaneous endocardial septal pulsed field ablation; TTE, transthoracic echocardiography; and 6MWD, 6-minute walk distance.

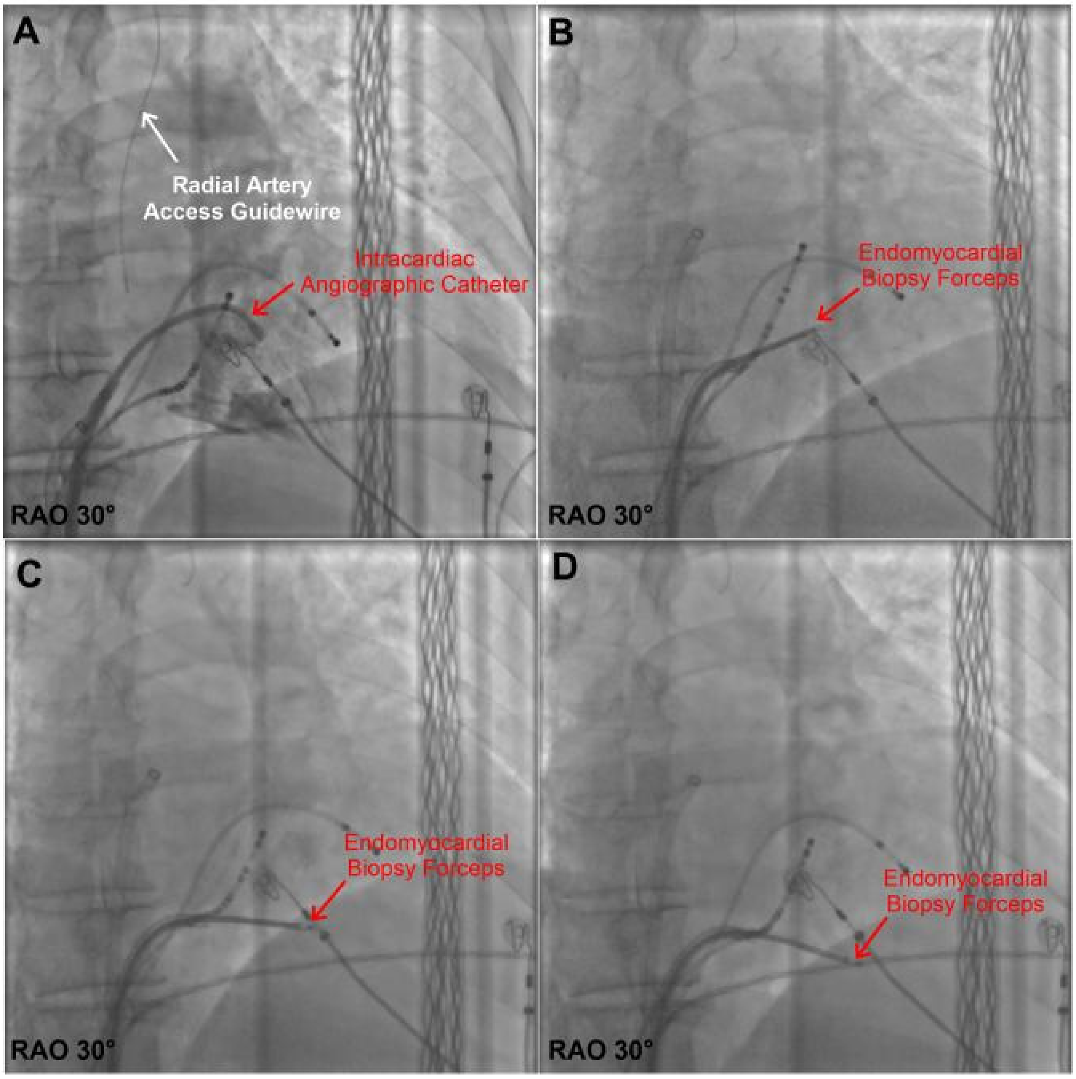


**Supplemental Figure S2. Fluoroscopic imaging of the endomyocardial biopsy procedure.**

(A) Right ventricular and pulmonary artery angiography performed with a 7-F angiographic catheter to identify the optimal site for endomyocardial biopsy.

(B–D) Endomyocardial biopsy specimens obtained with biopsy forceps via the right ventricle from the basal, mid, and apical portions of the interventricular septum, respectively.

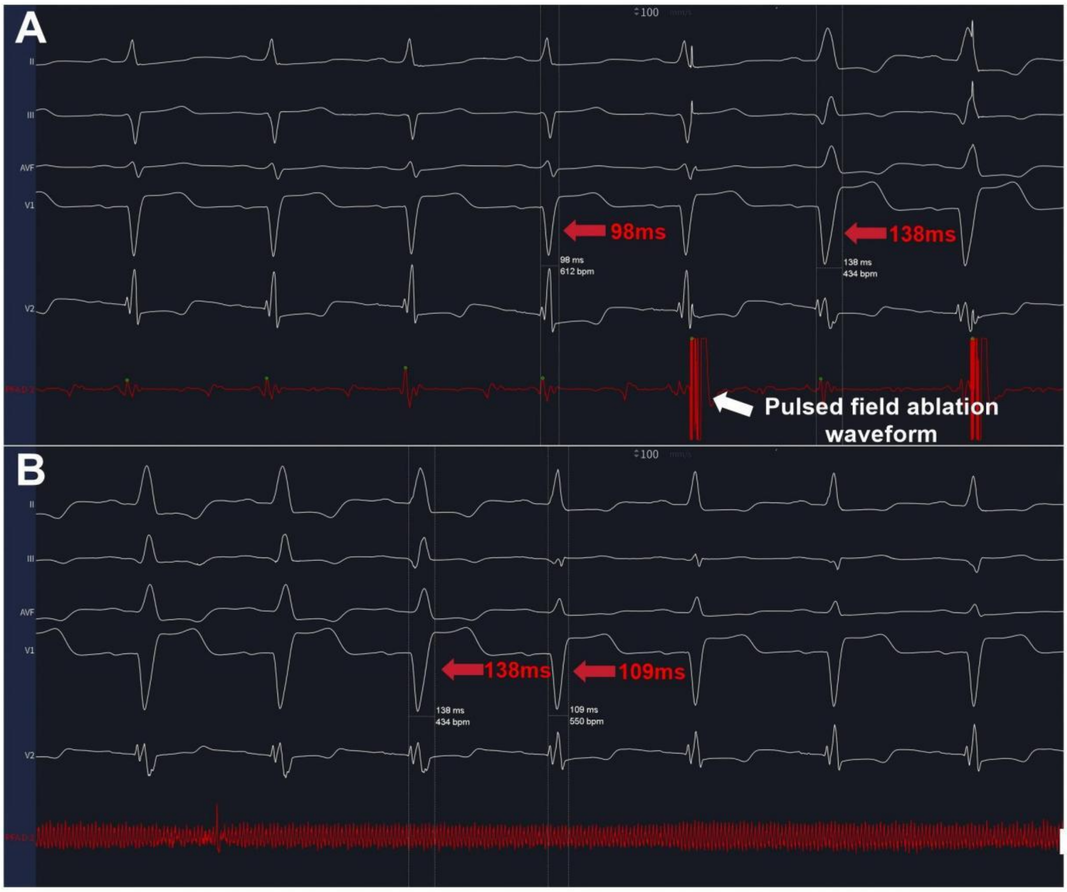


**Supplemental Figure S3. Transient left bundle branch block during PES-PFA in Patient 1.**

White tracings denote surface ECG recordings, with the Roman numerals on the left indicating the corresponding ECG leads. Red tracings denote intracardiac electrograms recorded from the distal tip of the PFA catheter.

(A) During definitive ablation at the 29th target site using a preset output voltage of 1800 V, acute ST-segment elevation in lead V1 and QRS widening consistent with left bundle branch block occurred after delivery of the first of 5 planned pulse trains. Energy delivery was immediately discontinued, and no additional pulse trains were delivered at this site.

(B) QRS narrowing began approximately 2 minutes after cessation of energy delivery, and the baseline QRS morphology was fully restored by 3 minutes without temporary pacing. Further PES-PFA was subsequently terminated.

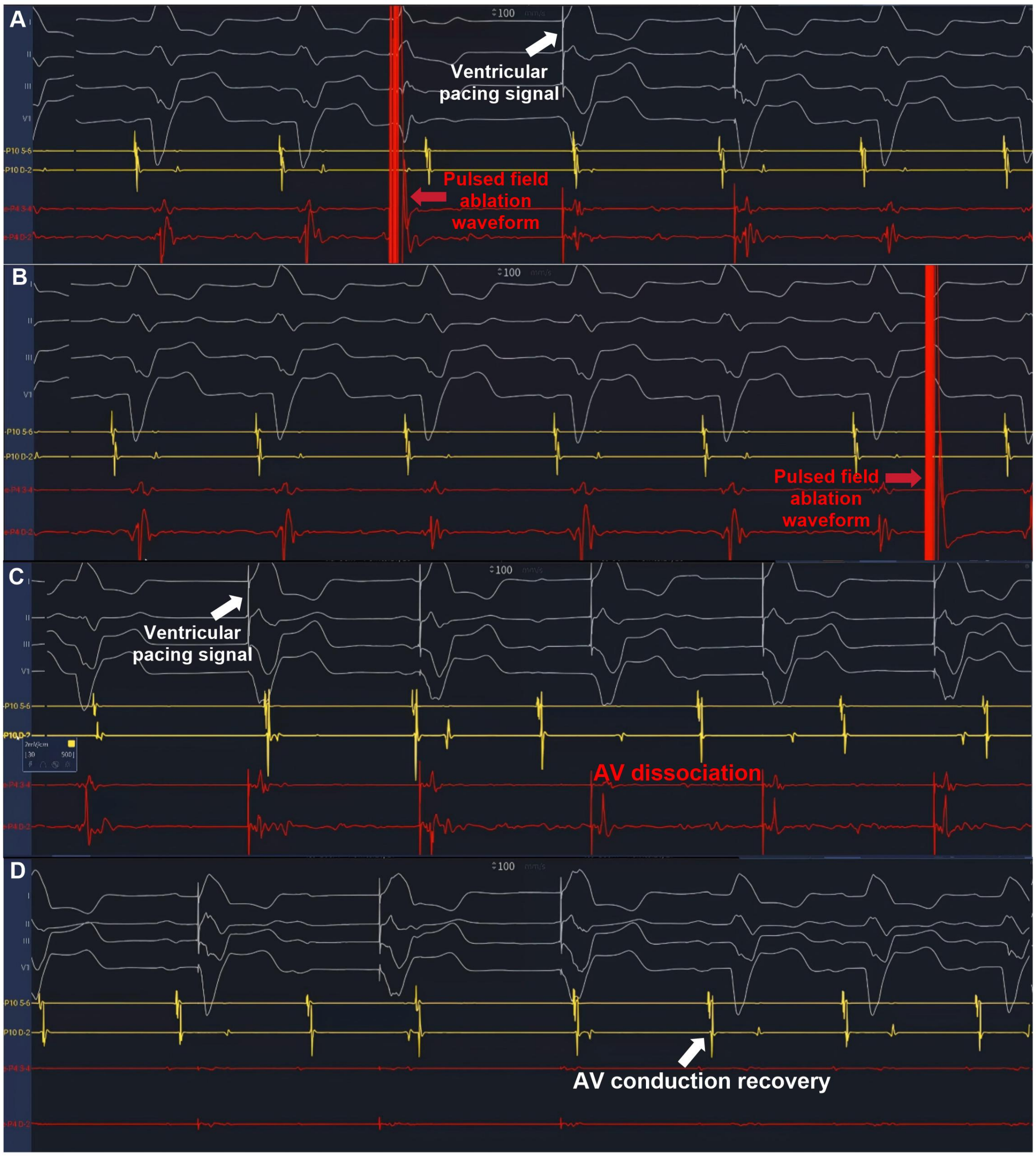


**Supplemental Figure S4. Transient complete atrioventricular block during PES-PFA in Patient 5.**

White tracings denote surface ECG recordings, with the Roman numerals on the left indicating the corresponding ECG leads. Yellow tracings denote coronary sinus electrograms, and red tracings denote intracardiac electrograms recorded from the distal tip of the PFA catheter.

(A) During low-dose test ablation at the 23rd target site using a preset output voltage of 800 V with 1 pulse train, 2 ventricular pacing signals were recorded immediately after energy delivery; intrinsic atrioventricular conduction then resumed without persistent conduction disturbance. The lower rate limit of the temporary pacemaker

was set at 60 beats/min.

(B) Intermediate-dose test ablation was subsequently performed at the same target site using a preset output voltage of 1600 V with 1 pulse train.

(C) Immediately after delivery of the pulse train, atrioventricular dissociation consistent with complete atrioventricular block occurred, accompanied by ventricular pacing backup signals.

(D) The ablation catheter was withdrawn, intravenous dexamethasone was administered, and ventricular pacing was maintained for approximately 4 minutes under continuous monitoring. Atrioventricular conduction gradually recovered approximately 4 minutes after onset. Further PES-PFA was then terminated, and pulmonary vein isolation was subsequently performed for atrial fibrillation. The temporary pacing lead was retained during pulmonary vein isolation and removed after completion of the entire index procedure.

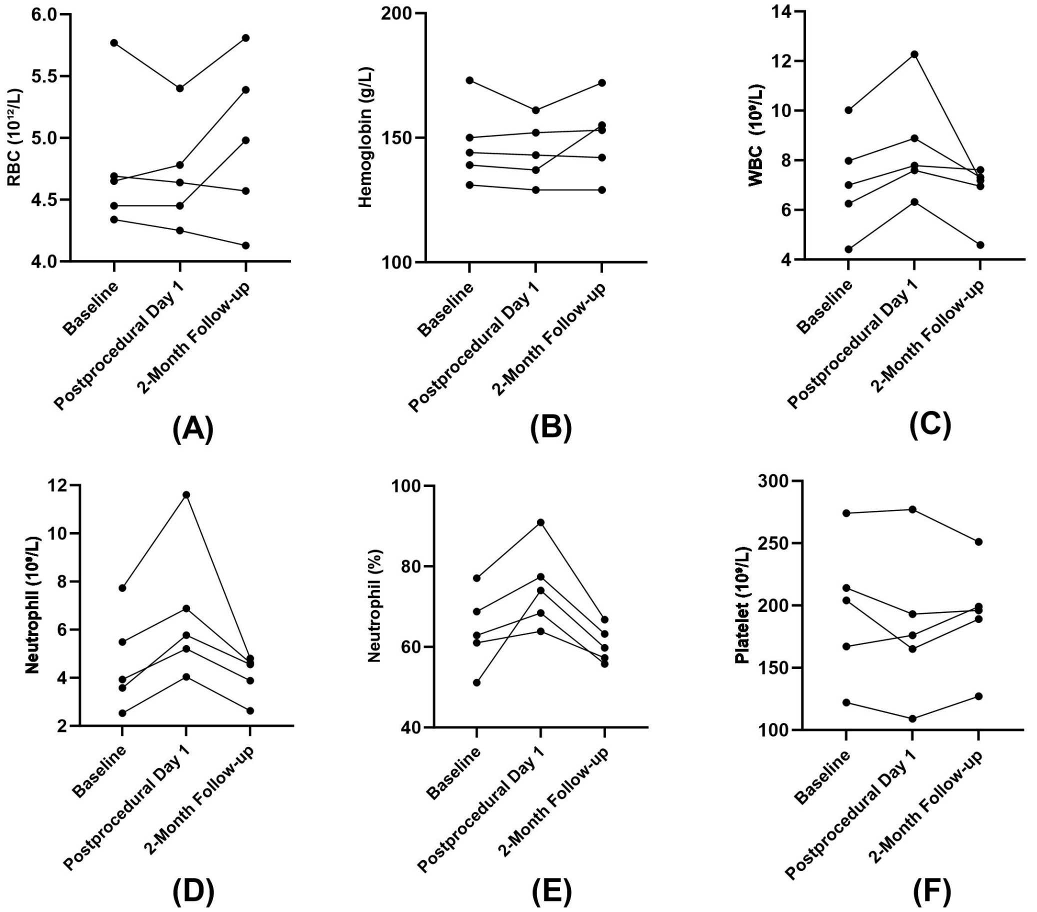


**Supplemental Figure S5. Periprocedural and 2-month changes in complete blood count parameters.**

(A) Red blood cell count.

(B) Hemoglobin level.

(C) White blood cell count.

(D) Neutrophil count.

(E) Neutrophil percentage.

(F) Platelet count.

Each line represents an individual patient. Measurements were obtained at baseline, on postprocedural day 1, and at 2-month follow-up.

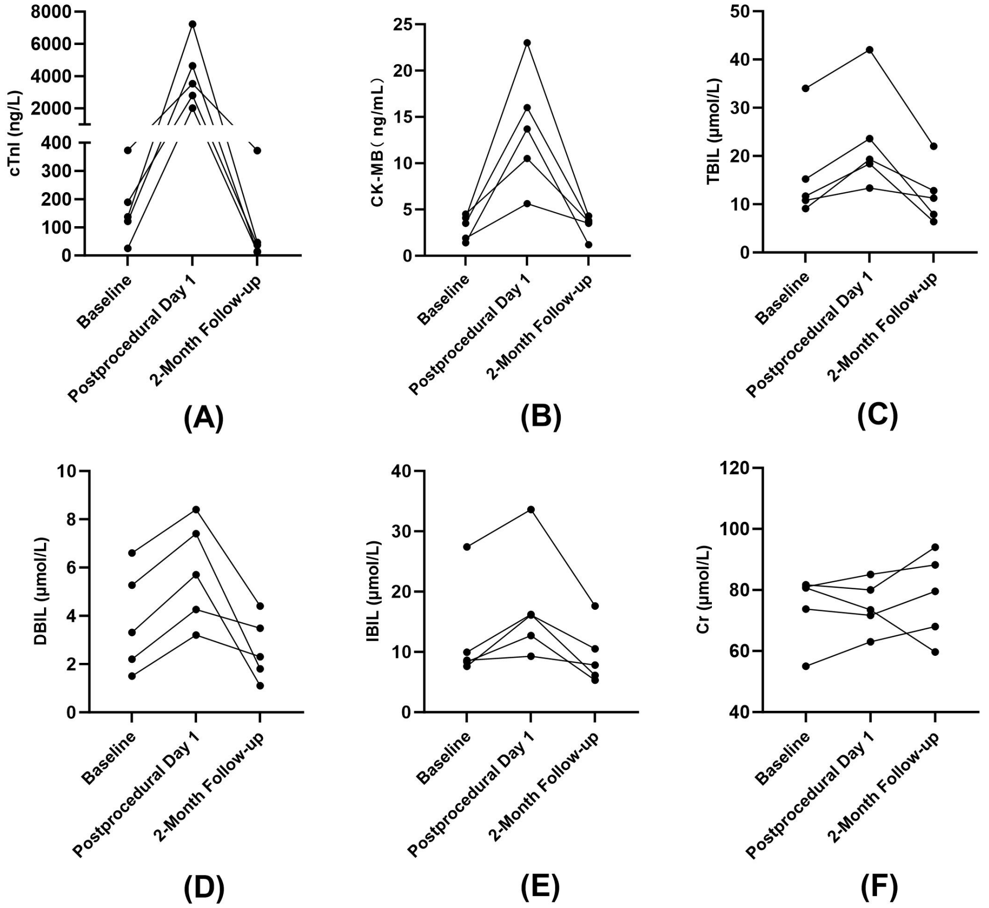


**Supplemental Figure S6. Periprocedural and 2-month changes in biochemical parameters.**

(A) Cardiac troponin I (cTnI).

(B) Creatine kinase-MB (CK-MB).

(C) Total bilirubin (TBIL).

(D) Direct bilirubin (DBIL).

(E) Indirect bilirubin (IBIL).

(F) Creatinine (Cr).

Each line represents an individual patient. Measurements were obtained at baseline, on postprocedural day 1, and at 2-month follow-up.

**Supplementary Note: Experimental Methods and Observations for Exploratory Porcine PFA**

One clinically healthy male Luchuan pig (5 months old; 45 kg) was used in this exploratory experiment, which was performed before initiation of the clinical case series. Supplemental Videos 1 and 2 show sequential stages of the experiment in the same animal; no additional animals underwent this exploratory conduction-system protocol. The animal was obtained from SINCE Biotechnology Inc. (Heilongjiang, China), acclimatized for at least 2 weeks under standardized conditions with a 12-hour light/12-hour dark cycle and an ambient temperature of 22–26°C, and fasted for 24 hours before the procedure. All procedures were approved by the Animal Ethics Committee of the First Affiliated Hospital of Harbin Medical University (approval No. 20241219) and complied with the National Institutes of Health Guide for the Care and Use of Laboratory Animals.

Anesthesia was induced with intramuscular Zoletil 50 (0.1 ml/kg; Virbac, France) and maintained with inhaled isoflurane delivered through a face mask with noninvasive ventilation. The animal was positioned supine, and heart rate, blood pressure, oxygen saturation, surface electrocardiography, and intracardiac electrograms were continuously monitored. After sterile preparation and local infiltration with 1% lidocaine, bilateral femoral venous access was obtained. A temporary pacing catheter was positioned at the right ventricular apex, and a controllable decapolar catheter (SinusFlex™, APT Medical, China) was positioned in the coronary sinus. A contact force–sensing PFA catheter (PulsedFA®, Jinjiang Electronic Medical, China) was used for mapping and ablation. Three-dimensional electroanatomical mapping and intracardiac electrogram recording were performed using the LEAD®-PTCA electrophysiology recording system (Jinjiang Electronic Medical, China).

Three-dimensional geometries of the right atrium, superior vena cava, and right ventricle were reconstructed. The superior vena cava region was identified anatomically on the reconstructed geometry. During sinus rhythm, activation mapping of the right atrium and superior vena cava was performed, and the earliest right atrial

activation area, displayed in red on the activation map, was designated as the presumed sinoatrial node region. The His bundle region was identified by recording distinct His potentials and was annotated with yellow points on the three-dimensional electroanatomical map. PFA was delivered at a preset output voltage of 1800 V using a biphasic waveform. Each site received one complete application consisting of 5 pulse trains delivered over 5 seconds, with no repeated energy application at any site. Pulse width and interpulse interval were proprietary and were not disclosed by the manufacturer. PFA was delivered sequentially in the superior vena cava region, within and surrounding the presumed sinoatrial node region, and at the mapped His bundle region.

PFA was completed at 14 sites in the superior vena cava region, 18 sites within and surrounding the presumed sinoatrial node region, and 1 site in the mapped His bundle region. Following PFA in the superior vena cava region, local electrical conduction block was observed, with no additional rhythm disturbance during the subsequent 3-minute observation period. Following PFA within and surrounding the presumed sinoatrial node region, no obvious change in sinus rate or apparent sinoatrial node dysfunction was observed during the subsequent 15-minute observation period. At the mapped His bundle site, one complete application at 1800 V was followed immediately by complete atrioventricular block. Dexamethasone sodium phosphate was administered intravenously at a dose of 5 mg every 15 minutes for 3 doses, for a cumulative dose of 15 mg, and temporary ventricular pacing was provided from the right ventricular apex at 60 beats/min. Atrioventricular conduction did not recover during the 1-hour observation period. The animal was subsequently maintained under deep isoflurane anesthesia. After absence of nociceptive and corneal reflexes was confirmed, the animal was euthanized by intravenous administration of sodium pentobarbital (100 mg/kg) in accordance with the approved protocol. Death was confirmed by the absence of spontaneous respiration, cardiac activity, arterial pulsation, and corneal reflexes. No histopathological assessment was performed in this exploratory experiment.

**Supplemental Video S1. Pulsed field ablation in the superior vena cava and presumed sinoatrial node regions in a porcine model.**

White points indicate the tricuspid annulus, yellow points indicate sites where His potentials were recorded, and red, light red, and green points indicate ablation sites. At each site, PFA was delivered at a preset output voltage of 1800 V as a single 5-second application consisting of 5 pulse trains; pulse width and interpulse interval were proprietary and were not disclosed by the manufacturer. PFA was completed at 14 sites in the superior vena cava region, after which local electrical conduction block was observed; no additional apparent rhythm disturbance was observed during the subsequent 3-minute observation period. PFA was completed at 18 sites within and surrounding the presumed sinoatrial node region, with no obvious change in sinus rate or apparent sinoatrial node dysfunction during the subsequent 15-minute observation period.

**Supplemental Video S2. Complete atrioventricular block induced by pulsed field ablation at the His bundle region in a porcine model.**

At a single site in the mapped His bundle region, one complete PFA application was delivered at a preset output voltage of 1800 V, consisting of 5 pulse trains over 5 seconds. White points indicate the tricuspid annulus, yellow points indicate sites where His potentials were recorded, and red, light red, and green points indicate ablation sites. Pulse width and interpulse interval were proprietary and were not disclosed by the manufacturer. This His bundle application was performed in the same animal after completion of PFA in the superior vena cava and presumed sinoatrial node regions. The complete 5-second application was followed immediately by complete atrioventricular block. Despite intravenous dexamethasone sodium phosphate administered at 5 mg every 15 minutes for 3 doses, for a cumulative dose of 15 mg, and temporary right ventricular apical pacing at 60 beats/min, atrioventricular conduction did not recover during the 1-hour observation period. After completion of the 1-hour observation period, the animal was euthanized under deep anesthesia in accordance with the approved animal protocol.
